# Metabolic therapy and bioenergetic analysis: The missing piece of the puzzle

**DOI:** 10.1016/j.molmet.2021.101389

**Published:** 2021-11-05

**Authors:** Tomás Duraj, Josefa Carrión-Navarro, Thomas N. Seyfried, Noemí García-Romero, Angel Ayuso-Sacido

**Affiliations:** 1 Faculty of Medicine, Institute for Applied Molecular Medicine (IMMA), CEU San Pablo University, 28668, Madrid, Spain; 2 Faculty of Experimental Sciences, Universidad Francisco de Vitoria, 28223, Madrid, Spain; 3 Brain Tumor Laboratory, Fundación Vithas, Grupo Hospitales Vithas, 28043, Madrid, Spain; 4 Biology Department, Boston College, 140 Commonwealth Ave, Chestnut Hill, MA, 02467, USA; 5 Faculty of Medicine, Universidad Francisco de Vitoria, 28223, Madrid, Spain

**Keywords:** Cancer, Energy metabolism, Glycolysis, Oxidative phosphorylation, Research design

## Abstract

**Background:**

Aberrant metabolism is recognized as a hallmark of cancer, a pillar necessary for cellular proliferation. Regarding bioenergetics (ATP generation), most cancers display a preference not only toward aerobic glycolysis (“Warburg effect”) and glutaminolysis (mitochondrial substrate level-phosphorylation) but also toward other metabolites such as lactate, pyruvate, and fat-derived sources. These secondary metabolites can assist in proliferation but cannot fully cover ATP demands.

**Scope of review:**

The concept of a static metabolic profile is challenged by instances of heterogeneity and flexibility to meet fuel/anaplerotic demands. Although metabolic therapies are a promising tool to improve therapeutic outcomes, either via pharmacological targets or press-pulse interventions, metabolic plasticity is rarely considered. Lack of bioenergetic analysis *in vitro* and patient-derived models is hindering translational potential. Here, we review the bioenergetics of cancer and propose a simple analysis of major metabolic pathways, encompassing both affordable and advanced techniques. A comprehensive compendium of Seahorse XF bioenergetic measurements is presented for the first time.

**Major conclusions:**

Standardization of principal readouts might help researchers to collect a complete metabolic picture of cancer using the most appropriate methods depending on the sample of interest.

## Highlights

• Normal and cancer cells differ in substrate level phosphorylation and mitochondrial rewiring of ATP-generating pathways.
• Glucose and glutamine are two major fuels for tumor growth. Their allocation is dependent on mitochondrial function.
• Secondary metabolites assist in proliferation but cannot fully cover ATP demands.
• Metabolic therapy is a promising cancer management strategy, but standardization and patient stratification is required.
• Bioenergetics can be assessed *in vitro* using bench top and integrated solutions and *in vivo* via metabolic imaging.

## Abbreviations

OXPHOS: oxidative phosphorylation
SLP: substrate-level phosphorylation
ETC: electron transport chain
FAO: fatty acid oxidation
mtDNA: mitochondrial DNA
OCR: oxygen consumption rate
KBs: ketone bodies
BHB: β-hydroxybutyrate
AcAc: acetoacetate
LDH: lactate dehydrogenase
BCAAs: branched-chain amino acids
ORR: optical redox ratio
TPEF: two-photon excited fluorescence
ECAR: extracellular acidification rates
MMP: mitochondrial membrane potential
CEB: Cell Energy Budget Platform
SCM-PAM: single-cell metabolic photoacoustic microscopy
PET: positron emission tomography
MRS: magnetic resonance spectroscopy
CT: computed tomography
MRI: magnetic resonance imaging
CEST: chemical exchange saturation transfer
BIRDS: biosensor imaging of redundant deviation in shifts
LC/GC–MS: liquid and gas chromatography-mass spectrometry
SIRM: stable isotope-resolved metabolomics
DGE: dynamic glucose-enhanced
DNP: dynamic nuclear polarization

## 1 Introduction

Study of cancer metabolism has seen a resurgence in recent years, with interest to guide therapies at the distinct metabolic characteristics of cancerous cells; this renewed focus is substantiated by the fact that altered metabolism has been integrated as one of the principal hallmarks of cancer [1].

Several theories about the metabolic origin of cancer have resulted in scientific debate due to their seeming confrontation with the somatic mutation theory [2]. Currently, research efforts are fundamentally directed at the study of genetic aberrations, with arguably limited cost-effectiveness and translational success [[2], [3], [4], [5]]. This has led to a call for major revisions in the molecular paradigm of cancer [2,5,6].

Almost a century ago, Warburg postulated a theory where all cancer cells arise from mitochondrial defects in structure and function, making them avid consumers of glucose and other fermentable sources, even in the presence of oxygen [7]. This metabolic phenotype was termed “Warburg effect” or “aerobic glycolysis”. Altered mitochondrial metabolism leads to variable dysfunction in oxidative phosphorylation (OXPHOS) [8,9]. In this model, genetic rewiring is a non-causal downstream epiphenomenon of mitochondrial failure, ROS-induced mutations and derailed inputs into primitive energy sensing pathways [10].

Instead of antagonizing genomic and mitochondrial contributions to tumorigenesis, metabolomics, proteomics, big data analysis, and mitochondrial respirometry can help us integrate conflicting results into a unified model. Notwithstanding the contentious origin problem, recent insights indicate a close connection between molecular and metabolic rewiring [11]. As a practical application, oncometabolic signatures from tumoral samples have been integrated into therapeutically relevant predictive models [12]. Unfortunately, the clarity of the accumulating evidence is obfuscated by a lack of systematic collection of relevant metabolic information.

In this review, we incorporated well-known and widely replicated experiments of bioenergetic metabolism as the backdrop to propel the need for standardized metabolic analysis. First, we describe all major metabolic pathways that lead to ATP production in normal and cancer cells. Second, we discuss the successes and pitfalls of metabolic therapies, emphasizing the lack of appropriate patient stratification as a key unresolved challenge. Third, we offer a summary of tools and techniques to obtain *in vitro* and *in vivo* measurements of bioenergetic metabolism. Seahorse XF technology is spotlighted as a widespread method for metabolic analysis that requires careful interpretation and experimental design.

We conclude that guiding research endeavors towards evolutionarily conserved bioenergetic or biosynthetic pathways could be a way of reducing molecular complexity and increasing the effectiveness of cancer therapies. A better characterization of tumoral and healthy samples could cast new light about cancer growth, designing comprehensive studies which include altered metabolism as a prominent driver of proliferation.

## 2 Master metabolic regulators

Despite the ample genetic and histological heterogeneity of cancer, a relatively small number of metabolic processes is responsible for maintaining ATP generation and redox balance [13]. ATP can only be physically generated in two distinct processes: substrate-level phosphorylation (SLP) or fueling the proton gradient of the mitochondrial electron transport chain (ETC). Their adequate functioning is essential for the maintenance of a proliferative status, redox balance and cell viability [14].

**Figure 1:**
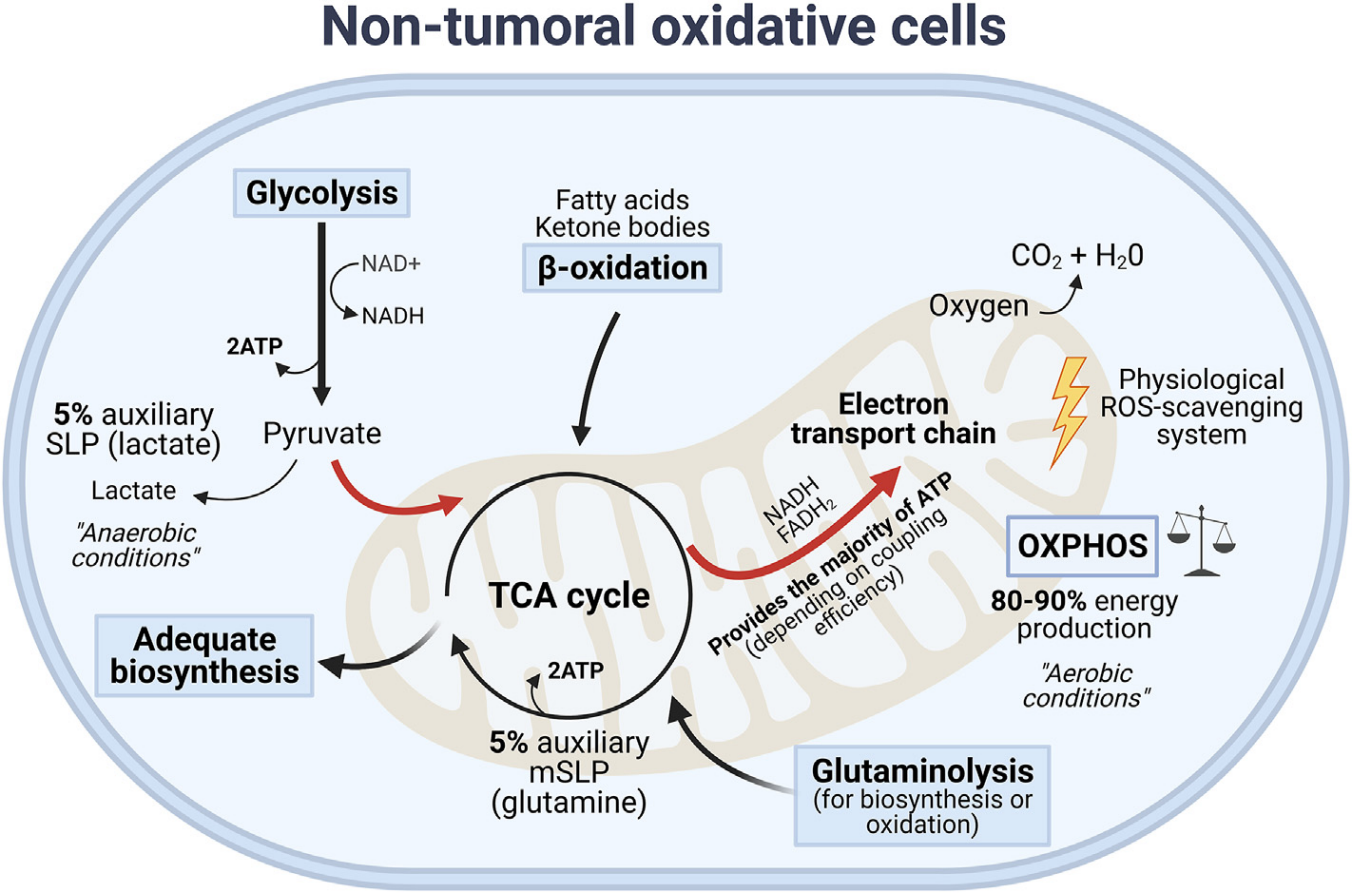
**Simplified diagram of the distribution of fuels in non-tumoral, oxidative cells.** OXPHOS is estimated to provide at least 80% of ATP under conditions of oxygen abundance, generating a manageable amount of ROS. SLP-ATP generation is upregulated in anaerobic conditions. This diagram is intended to serve as a point of comparison between nontumoral and transformed cells, but it does not encompass all the dynamic properties of metabolic regulation in physiological conditions.

As shown in Figure 1, healthy cells exhibit efficient, fine-tuned bioenergetic metabolism. Under physiological conditions, glycolysis is the cytosolic conversion of glucose into two molecules of pyruvate, which are then oxidized in the mitochondria, yielding ≈30 ATP, CO_2_ and H_2_O per glucose molecule [15]. In most normal cells, glucose-driven OXPHOS entails the primary mechanism for energy production [16]. However, in the absence of oxygen (anaerobic glycolysis), pyruvate can be enzymatically reduced to lactic acid, generating a net total of 2 ATP.

In cancer cells, the persistent production of lactate from pyruvate, regardless of the presence of oxygen and forgoing mitochondrial oxidation, is known as “aerobic fermentation,” “aerobic glycolysis,” or “Warburg effect.” Catabolic fuels (glucose, glutamine, pyruvate, lactate, free fatty acids, ketone bodies, cysteine, serine, and other amino acids) can be obtained from circulation, stroma, *de novo* synthesis, and/or autophagy within the cell itself [17,18]. Tumors are metabolically heterogeneous and different cell populations can express preferences toward specific microenvironmental metabolites [19].

**Figure 2:**
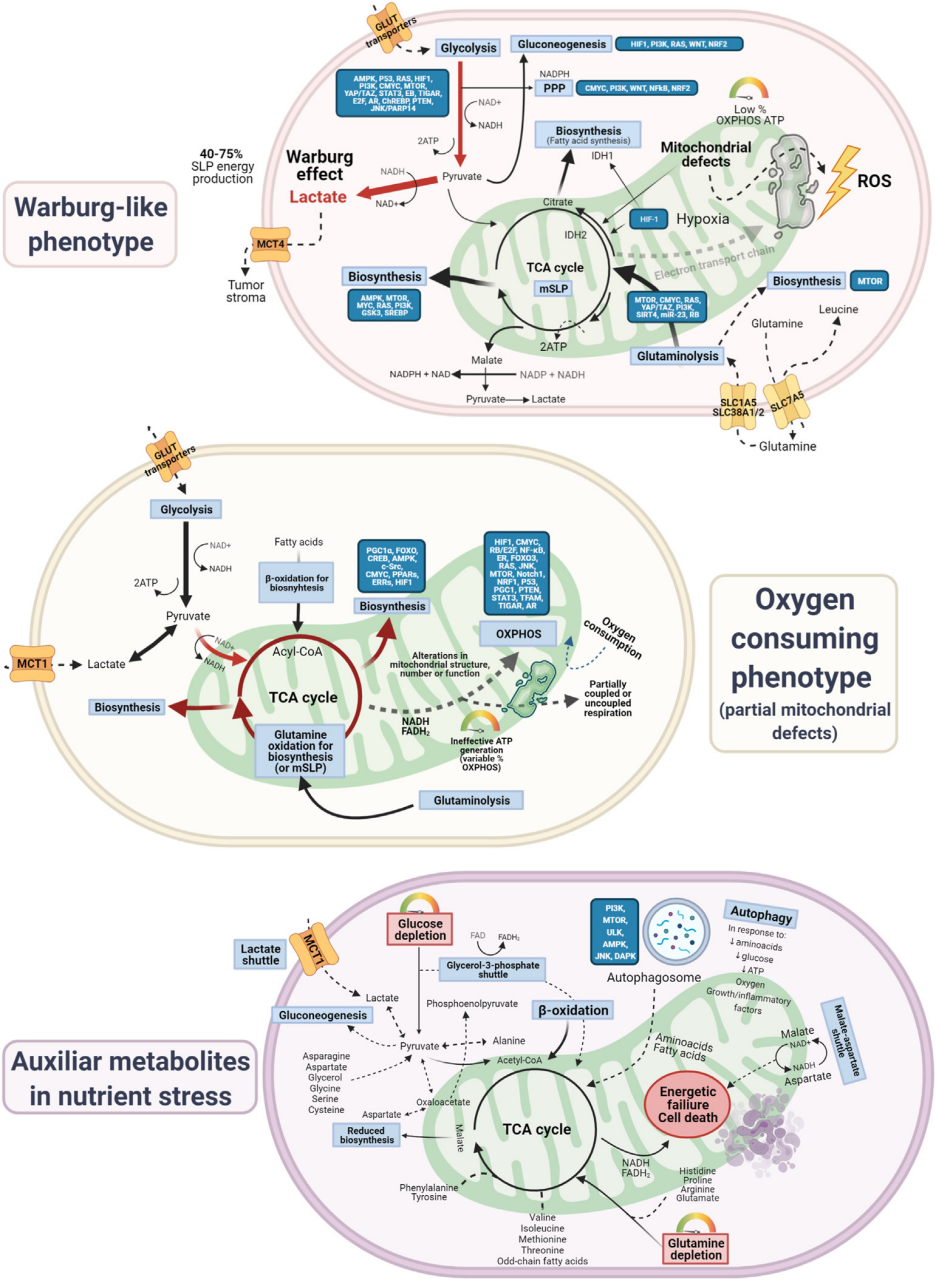
**Tumoral bioenergetic metabolism at a glance.** Glucose enters transformed cells via GLUT family transporters and undergoes glycolysis, converted into pyruvate (yielding 2 net ATP). Cancer cells preferentially divert pyruvate to lactate (SLP), regardless of the presence of oxygen (aerobic glycolysis or Warburg effect); an estimated 10% of pyruvate goes toward biosynthesis, and OXPHOS is variable, depending on mitochondrial dysfunction. Oxygen consuming, mitochondrially defective cancer cells can utilize glucose-derived pyruvate, glutamine, and fatty acids to drive TCA cycle intermediaries, but ATP-generation capacity through the ETC is impaired to a substantial degree. In conditions of glucose and glutamine deprivation, recycling of ancillary substrates could aid in ATP generation, including fatty acids/ketone bodies, lactate, amino acids and autophagic processes. Metabolic reprogramming involves the interconnected regulation of glycolysis, glutaminolysis and mitochondrial metabolism. Prominent regulators are *P53* (glycolysis/OXPHOS), *CMYC* (glycolysis, glutaminolysis and lipid synthesis), *KRAS* (glycolysis, glutaminolysis), *PI3K/AKT* (glycolysis), *AMPK* (nutrient stress), *HIF-1* (glycolysis, global metabolic rewiring), peroxisome proliferator-activated receptors (*PPARs*) and PPARγ coactivator-1α (*PGC-1α*) (FAO), forkead box O family (*FOXO*) (OXPHOS). The combination of preexisting oncogenic lesions and metabolic fluxes, along with tumoral milieu, is likely to govern metabolic flexibility of each individual cell. Abbreviations: PPP (Pentose phosphate pathway), SLP (substrate level phosphorylation), OXPHOS (oxidative phosphorylation), ROS (reactive oxygen species).

Warburg-like and oxygen consuming phenotypes have been extensively described in cancer as both discreet and plastic categories, as summarized in Figure 2. Oxygen consumption, however, cannot be interpreted as functioning ATP generation at the mitochondrial level due to ample evidence of mitochondrial defects in almost all subtypes of cancer [9]. Despite the supporting role of other metabolites, cancer cells are largely dependent on glucose and glutamine as either fermentable or oxidative fuels. The reach and scope of metabolic flexibility, that is, the ability of any given cancer cell to shift between metabolic states, remains unclear.

## 3 Catabolic pathways contributing to ATP synthesis

### 3.1 Warburg effect: selfish, rapid, and oxygen-independent ATP production to fuel proliferation over bioenergetic efficiency

The Warburg effect embodies perhaps the single most studied and misunderstood metabolic alteration of cancer [20]. An unresolved debate stands about its independence from the microenvironment, mitochondrial fitness, and sufficiency/necessity for malignant transformation [21]. It was hypothesized that this metabolic switch might be a preemptive adaptation to variations in intratumoral blood flow by improper angiogenesis [22]. In the endosymbiotic mitochondrial model, the Warburg effect signifies a regression to an evolutionary conserved glycolytic, proliferative phenotype, triggered by compromised mitochondrial integrity/function [23]. Speed of energy production is another reason to consider SLP an advantageous, rapidly adjustable metabolic state: although the amount of ATP per mole of substrate produced by OXPHOS is higher, aerobic glycolysis is almost 100 times faster [24]. Awareness about the predominance of the Warburg phenotype in most cultured cells should not be an excuse to underestimate its relevance, especially when outlining cancer as unchecked proliferation with a primary metabolic component.

Glucose is avidly consumed by most cancer cells through metabolic rewiring to compensate for the low ATP generation yields due to mitochondrial defects as well as the predominance of the glycolytic PKM2 isoform, which produces intermediary metabolites rather than ATP [25,26]. Glucose is destined mainly for fermentation and biosynthetic processes, not OXPHOS. To date, Warburgian phenotypes had been observed in almost all solid tumors [16,27]. Counterpoint evidence for a more nuanced metabolic heterogeneity was suggested in oxygen-consuming phenotypes [28], but it is important to emphasize that extracellular measurements of oxygen do not translate to ATP synthesis by the ETC. As ATP levels in cancer cells are still maintained under irreversible mitochondrial inhibition, normal OXPHOS cannot occur in the majority of cancer cells [29,30]. As a juxtaposition, a hybrid model of metabolic clustering termed the “reverse Warburg effect” or “Autophagic Tumor Stroma Model” has been put forward to describe the interactions between cancer cells and tumoral stroma [[31], [32], [33], [34], [35]]. Lactate is an important source of TCA cycle intermediates in physiological conditions and should be recognized as a potential target in cancer, regardless of the discussion surrounding the oxidative fitness of cancer cells [36,37]. While ancillary metabolites could support tumor growth, especially for biosynthetic purposes, as discussed below, they cannot drive sufficient ATP synthesis in conditions of glucose and glutamine depletion [38,39]. Furthermore, glucose uptake is not necessarily destined for fermentation or oxidation, but can be diverted mostly to anabolic pathways [40].

Mitochondrial integrity and ATP-generating capacity could be understood as the underlaying factor that dictates the fate of glucose-derived pyruvate and metabolic heterogeneity. A more appropriate way to conceptualize the Warburg effect might be a spectrum, rather than a binary switch, with cancer cells displaying unique glucose and glutamine dependencies based on their degree of mitochondrial dysfunction, tissue of origin, and ROS-induced mutational landscapes [41]. While accurate measurement of mitochondrial function has not been accomplished in all models of cancer, it can elegantly explain apparent differences in metabolic phenotypes without the need to characterize each individual effector-gene, rewired in a non-causal manner. As a solution to this methodological issue, a wide variety of techniques to measure mitochondrial metabolism are highlighted in our review.

### 3.2 Oxidative phosphorylation: mitochondrial fitness as the arbitrator of respiration

OXPHOS produces energy through the transfer of electrons from high-energy electron carriers (NADH or FADH2) to a series of mitochondrial complexes located in the inner membrane and by the generation of a proton gradient. The resulting electromotive force converts ADP to ATP via ATP synthase. At the same time, redox carriers derive from cytoplasmatic glycolysis, tricarboxylic acid (TCA) cycle and fatty acid oxidation (FAO). The term OXPHOS itself is substrate-agnostic, although the majority of electron donors originate from glucose oxidation in normal cells [42], and between 25 and 60% in cancer cells, with a high degree of variability between subtypes [43].

Cancer cells have evidence of abnormalities in mitochondrial structure, biogenesis, and mitochondrial DNA (mtDNA), together with reduced activity of ETC complexes [29,[44], [45], [46], [47], [48]]. Normal cristae shape is essential to evaluate ATP synthesis, as structure and function cannot be separated [49,50]. Mitochondrial transfer studies corroborated that normal mitochondria can inhibit tumorigenesis and reverse the Warburg effect, even when coexisting with a tumorigenic genome [27,51,52]. Conversely, a compelling demonstration of the importance of mitochondrial regulation for neoplastic transformation comes from cancer cells lacking mtDNA, that required mitochondrial acquisition from neighboring cells to efficiently generate tumors *in vivo* [53]. In both healthy cells and cancer cells, mitochondria regulate cell proliferation, metabolic adaptation, and Ca_2_^+^ homeostasis [54]. Metabolic reprogramming enables proliferation despite varying degrees of mitochondrial dysfunction [[55], [56], [57]]. As a merely descriptive exercise, when OXPHOS decreases, SLP must increase to ensure constant ATP supply; this interplay has been replicated in direct measurement studies at the cellular level [58] and simulated in flow balance and mathematical models [59]. Nonetheless, the inhibition of glycolysis also increases other processes such as glutamine-dependent SLP/OXPHOS [60].

The relevance of mitochondrial alterations is still unclear, as definite determination of function without relying on indirect measurements, such as oxygen consumption rates (OCR), is a challenging endeavor [61].

Contemplating glycolytic capacity as a survival advantage and, at the same time, a vulnerably due to defective mitochondria are two opposing concepts [62]. Contrary to Warburg's hypothesis, a relative dependence upon oxygen-consuming phenotypes has been described in specific cancer subtypes [63,64]. Even still, as indicated by metabolic profiling (described in subsection 5.1 and Table S2), cancer cells in which OCR is the predominant state are very rare, always coexisting with glycolytic compensation. Beyond ATP synthesis, identification of oxidative metabolism could be applied as a predictor of chemo-resistance [65]. OCR, measured by a variety of respirometry techniques, differed greatly between and within the same cell lines, further confused by cell handling and normalization issues [66]. To the extent of our knowledge, no experimental evidence of cancer cells sustained exclusively and self-sufficiently by oxidation of glucose, glutamine, or fatty acids has been described (Table S3). To which extent is OCR representative of healthy mitochondrial function is being questioned: it cannot detect glutamine-driven mitochondrial SLP (mSLP) or allocate any other metabolic fuels to their final destination; thus, measuring OCR alone should not be considered an unequivocal marker of functioning respiration [29,67].

### 3.3 Glutaminolysis: mitochondrial substrate-level phosphorylation and anaplerosis

Glutamine is a conditionally essential α-amino acid involved in cell proliferation, tumor growth, and nucleotide synthesis [68,69]. As an important anaplerotic source, it can be metabolized into glutamate and, subsequently, α-ketoglutarate, to enter the TCA cycle and drive biosynthesis and OXPHOS by generation of electron cofactors. Glutaminolysis can generate 2 ATP molecules through succinyl-CoA synthetase in a enzymatic reaction called mSLP [70], which is believed to generate substantial amounts of ATP in the absence of glucose, under hypoxic conditions, or when mitochondrial integrity is compromised [[71], [72], [73]].

Moreover, mSLP could be erroneously interpreted as a marker of mitochondrial fitness using flux analyzers, whereas stable isotope tracing studies demonstrated its role in response to moderate (catabolic) or severe (anabolic) OXPHOS dysfunction [74]. Under hypoxic conditions, glutaminolysis has been shown to produce most of the ATP and synergize with the Warburg effect to promote tumor growth [75]. Addiction to glutamine has now been documented in almost all subtypes of cancer, including lung, ovarian, pancreatic, colorectal, breast, and brain tumors, with metabolic reprogramming (principally by the *MYC* family of genes) for increased anaplerosis, regulation of nitrogen balance, redox homeostasis, and mSLP [76]. Because the contribution of glutamine is fluctuating and not fully characterized in all model systems, the absolute contribution of glucose and glutamine oxidation/SLP to ATP synthesis for any given cell line is largely unknown [59,77].

In cancer, glutamine-dependent reductive carboxylation becomes a major pathway of citrate formation for lipid synthesis, generation of TCA metabolites, and macromolecular precursors [56]. In addition to roles as a carbon/nitrogen biosynthetic source [78,79], glutamine has been implicated in enzymatic release of ammonia into the microenvironment [80]. Free ammonia can be recycled into central amino acid metabolism to maximize nitrogen utilization [81,82]. A participation in acidic protection was touted through enzymatic deamidation [83]. Cancer cells are likely able to acquire sufficient quantities of glutamine from circulation with increased blood levels reported in advanced stage cancers [84]. *De novo* synthesis of glutamine could be sufficient to compensate glutamine starvation via glutamine synthetase and rescued by other amino acids, particularly asparagine, aspartate, and arginine [85,86]. Although less understood from a cancer perspective, glutamine has been implicated in the regulation of autophagy [87,88].

Lastly, *in vitro* studies are generally performed under conditions of glutamine abundance to improve proliferation rates, thus reducing translational potential [89]. Likewise, levels of metabolites inside the tumor mass are variable: for example, colon, stomach, and oral cancers presented low glucose and glutamine levels with high lactate and glutathione, suggesting Warburg-like properties [90]. Synergistic inhibition of glycolysis and glutaminolysis is proposed as a promising therapeutic approach [91]. In clinical trials, metabolic flux analysis and stratification of patients according to glutaminolysis will be crucial to improve responses to both conventional therapy and metabolic drugs.

### 3.4 Fatty acid oxidation and ketone bodies

Saturated and unsaturated fatty acids can be oxidized for energy via medium and long-chain acyl-CoA dehydrogenases and β-oxidation input into the TCA cycle, generating acetyl-CoA and electron donors. FAO yields 2.5 times more ATP per mole of substrate than glucose (between 14 and 17 ATP every oxidation cycle) [92]. Elevated levels of FAO have been detected even in abundance of glucose and glutamine [93]. When the AMP/ATP ratio increases, AMPK activates fatty acid metabolism to provide catabolic substrates, maintain redox homeostasis, and biosynthesis. In hypoxia, FAO is suppressed by *HIF-1* [94,95]. Even though specific cell lines (e.g., ovarian, prostate, breast, brain, and colon) demonstrated relatively higher reliance upon FAO, complete ATP provision from this pathway without substantial contribution of glycolysis/glutaminolysis is uncommon [96]. Rather, lipid metabolism might contribute to redox balance and biosynthetic pathways, while also regulating glycolysis and glutaminolysis [97,98].

Ketone bodies (KBs), β-hydroxybutyrate (BHB) and acetoacetate (AcAc), and the short-chain fatty acid acetate, have been identified as non-fermentable substrates. KBs metabolism is especially relevant in metabolic therapies based on glucose/glutamine inhibition, where rescue of healthy cells via BHB has been proposed as an adjuvant therapeutic strategy [99,100]. In humans, acetate can be a secondary energy source, thought to play a very minor role in tumor growth, with very low *in vivo* levels [101]. Although tumor cells can express ketolytic enzymes, the argument has been made that most mitochondrially defective cells struggle to process fats and KBs for fuel, especially in conditions of low glucose [102,103]. It was also shown that hypoxia and nutrient stress can increase the dependence of some cancers upon FAO and KBs [104,105]. KBs generally possess antitumoral properties, but, under certain conditions, might promote cancer stemness [106,107]. Instead of relying on single pathway inhibition, combined press-pulse strategies might be the key to improve therapeutic responses [99].

In conclusion, KBs and fatty acids can be supplied either from circulation, autophagy, or adjacent stromal cells [108]. The importance of fat-derived sources to strictly bioenergetic production is heterogeneous and largely unspecified [59]. Identifying cancers with an abnormally high affinity for such obligate-OXPHOS catabolites is an appealing proposition for adequate patient stratification.

### 3.5 Ancillary metabolites and pathways: lactate and pyruvate in the reverse Warburg effect, anaplerotic amino acids and autophagy

Monocarboxylates have been examined extensively as central ATP-generating molecules [109,110].

Lactate, a downstream metabolite of pyruvate via lactate dehydrogenase (LDH), can itself act as a secondary energy source for tumor cells. Lactate can be synthetized independently of hypoxia and supplied by tumoral stroma [111,112]. Lactate can be derived to both alanine and glutamate synthesis [113,114]. In the two-compartment model of cancer metabolism, glucose fermentation leads to lactic acidosis, promoting a transition to oxidative metabolism in surrounding, well irrigated, oxygenated cells [115]. This assumes full metabolic flexibility of both cancer cells and associated fibroblasts, which might be limited to specific models and not representative of tumors with mitochondrial dysfunction [116,117]. High levels of lactate can partially rescue from nutrient depletion, but most cancer cells cannot proliferate with lactate alone [31,118]. Owing to the intrinsic regulation of glycolytic and mitochondrial metabolism, reversal of lactic acidosis showed promising results in preclinical and pilot human studies [119,120]. Lactate acts as a pro-metastatic metabolite, with functions in extracellular matrix remodeling through hyaluronan synthesis, enhanced cell motility, upregulation of *VEFG* and *HIF1* signaling, and immunomodulatory effects [109,121].

The monocarboxylate pyruvate could be viewed independently, originating primarily from glycolysis but also TCA sources (malate), amino acids (serine, cysteine, threonine/glycine and alanine), as well as lactate itself by reversal of LDH [122,123]. Pyruvate constitutes a central metabolic junction that can lead to the production of lactate, alanine, oxaloacetate, or acetyl-CoA [38]. Acetyl-CoA can remain in the TCA cycle or be metabolized in the cytosol for *de novo* lipogenesis, protein acetylation, or KB production [124]. Exogenous pyruvate has been shown to enhance invasive phenotypes: pyruvate oxidation correlates with proliferation and malignancy in some cancer cells [125]. Consequently, targeting pyruvate upstream or downstream is essential for the design of metabolic therapies, as pyruvate stands as an arbitrator between glycolysis and OXPHOS [126].

Complementary amino acids (methionine, threonine, phenylalanine, tyrosine, asparagine/aspartate, histidine, proline, arginine, and glutamate), especially branched-chain amino acids (BCAAs) isoleucine and valine, can also enter the TCA cycle at different steps as anaplerotic sources [127,128]. BCAAs can be metabolized by transaminases in both the cytosol and mitochondria, but their contribution to cancer progression is unclear [129]. Anaplerotic intermediaries might exert a partial rescue effect on tumor proliferation [130]. In this process, cysteine plays a pivotal role, being incorporated into glutathione, serving as a carbon source for energy metabolism and biosynthesis (via folate and methionine cycles), and contributing to the ETC as an indirect electron donor via hydrogen sulfide [131]. Alternatively, serine, glycine, folate oxidation, alanine, succinate, and ammonia, as well as other minor metabolites (glycerol, propionate), could aid in the survival of cancer cells by strengthening their metabolic flexibility in response to glucose/glutamine depletion [[132], [133], [134], [135]].

It should be noted that metabolic substrates are not imported exclusively from circulation or surrounding tissue, as autophagy, vesicular transport, and lysosomal digestion act as secondary sources [136,137]. Autophagy is a dynamic degradation process of cytoplasmic cellular self-constituents, allowing for macromolecule turnover even in a state of diminished nutrients (amino acids/glucose) and oxygen availability [138].

The question remains whether these supplementary metabolites, in quantities realistically achieved *in vivo*, be it from circulation, stroma, or autophagic processes, would be adequate to sustain tumor growth under synergistic targeting of glucose/glutamine. Thus far, it has been well established that glucose/glutamine are the two major bioenergetic and anaplerotic contributors, allowing adaptation to unfavorable microenvironment changes in oxygen and acidity by dynamic tuning of metabolic pathways [38,139,140].

## 4 Metabolic flexibility and therapy in the era of precision medicine

The incredible complexity of the genetic landscape and flow of information among genome, metabolome, and tumor microenvironment should be recognized as an important barrier for the translational success of molecular research [141]. One of the main limitations of applying metabolism-based strategies is the heterogeneity of cancer cells and their adaptive mechanisms against nutrient deprivation. In this paradigm, each individual cancer cell could have a unique metabolic profile [142]. Multiple inputs into the TCA cycle, in an everchanging microenvironment, compose a metabolic framework that is far more diverse than limiting our perspective to “glycolysis, glutaminolysis, and FAO”. As with genetic heterogeneity, addressing these metabolic differences individually appears almost unmanageable.

In the reverse Warburg effect hypothesis, transformed cells (preserved OXPHOS, anabolic, stem-like) and adjacent fibroblasts (glycolytic, catabolic) coexist [[143], [144], [145]], whereas in the mitochondrial theory of cancer, this cooperative link is not relevant for mitochondrially impaired cancer cells [146]. Autophagy, mitochondrial dynamics, and metabolic coupling have been described as possible contributors to tumor growth, but they have never been shown to produce sufficient ATP to sustain proliferation on their own [63,111,147]. This dual metabolic model is only appliable to tumors with important stromal components [145,148]. Integrating new findings to determine whether glycolysis and OXPHOS can co-occur or are mutually exclusive is under active investigation [[149], [150], [151]].

In conclusion, a comprehensive metabolic analysis of neighboring tissues should be recommended [112]. Categorization of glycolysis/OXPHOS as “predominant” phenotypes would be a way to by-pass the heterogeneity of the molecular landscape, but additional studies to fully characterize metabolic flexibility are indispensable. Therapeutic targeting will require synergistic approaches to ensure single-pathway inhibition cannot be rescued.

### 4.1 The promises and limitations of metabolic therapy: reconsidering standardization and heterogeneity

Metabolic therapy has been proposed as a promising management strategy for cancers with very poor prognosis and ineffective standard of care. Glycolytic and glutaminolytic dependencies are a well-described and targetable feature of many tumors, such as brain, pancreatic, breast, lung, gastric, skin, and prostate, among others [[152], [153], [154]]. Multiple interventions were suggested under the metabolic therapy umbrella, e.g., calorically restricted ketogenic diets, fasting, tumor microenvironment and oxidative stress regulation, hyperbaric oxygen therapy, hyperthermia, controlled hypoglycemia, autophagy inhibition, metabolic reprogramming, and inhibitors [99,[155], [156], [157], [158], [159]]; however, standardization is needed.

As a good candidate for metabolic therapy, high-grade gliomas have glycolytic phenotypes [160,161] and partial mitochondrial defects [29,162]. Mitochondrial inhibitors (such as metformin or IACS-010759) epitomize an attractive opportunity to specifically increase mitochondrial stress in cancer cells, while concurrently targeting glycolysis by dietary or pharmacological interventions [163,164]. Inhibiting OXPHOS and mitochondrial dynamics requires careful dosing and tissue localization to avoid off-target toxicity, being only intended to manage tumoral subpopulations with a partially functioning chondriome [165]. Stratification of mitochondrial dependencies in different cancer models and individual patients would be essential prior to synergistic SLP-OXPHOS targeting. Ketogenic compensatory strategies could protect healthy neuronal tissues against glucose deprivation [166,167]. Despite identification of Warburg-like vulnerabilities in glioma, metabolic heterogeneity needs to be incorporated into therapeutic designs [[168], [169], [170]].

In the same way, pancreatic cancer is an appealing target for metabolic intervention due to specific metabolic subtypes, *KRAS* governance, mitochondrial defects, and generally poor prognosis [171,172]. Accordingly, tumors of this origin have been classified into metabolic categories, with significant differences in glucose, glutamine, and mitochondrial metabolism, as well as a stromal component (reverse Warburg effect), highlighting the need for improved stratification [173,174].

Other solid tumors with broader metabolic plasticity are breast and lung cancers, where anti-glycolytic targeting has been proposed as a first-line therapy, but additional tailor-made approaches might be necessary to target all possible metabolic phenotypes [175,176]. In breast cancers, Warburgian/OCR phenotypes have been shown to coexist, thus requiring synergistic approaches to manage glycolysis and reverse mitochondrial dysfunction [177,178]. Lung cancers integrate oncogenic lesions, stromal contributions, and basal metabolic states into Warburg-like, glutaminolytic, mitochondrial, and mixed dependencies (lactate, serine, fatty acids, autophagy) [175,179,180].

In summary, owing to the proposed universal nature of altered metabolism, metabolic therapy would encompass all tumors with obligate SLP-ATP generation and defects in mitochondrial metabolism [181,182]. However, the identification of tumors characterized by a higher reliance on oxidative metabolites (e.g., glutamine or fat-derived), not exclusively for ATP synthesis but also other proliferative requirements, could be useful for stratification [41,183].

Unfortunately, initial excitement in this field has been met with difficulties to translate preclinical research into advanced human trials. Metabolic interventions are being evaluated predominantly in phase I and II pilot studies (e.g., dichloroacetate, 2-deoxyglucose, metformin, ketogenic diets, fasting, hyperbaric oxygen), with mostly favorable and encouraging outcomes [100,184]. Case reports and pilot clinical trials are a promising first step to introduce metabolic therapy into clinical practice [182,[184], [185], [186]]. Phase III clinical trials focus almost exclusively on metabolic imaging, with only a handful of trials assessing concrete therapeutic interventions, such as metformin (NCT02201381, NCT03031821), sirolimus (NCT04775173), fasting-mimicking diets (NCT02126449), and dietary supplements/life-style interventions to improve quality of life. Only one phase IV metabolic study is underway with metformin (NCT04741204).

As discussed, metabolic flexibility should be assessed in a case-by-case basis, and easy-to-use tools for bioenergetic analysis are finally becoming accessible for researchers. In translational studies, however, metabolic profiling is still relatively unheard-of. Metabolic plasticity and heterogeneity are also key factors in treatment response and resistance to standard chemoradiotherapy, but even with this knowledge, such concepts are often overlooked in experimental designs unless specifically testing metabolic agents [187].

## 5 Measurement of metabolic function: a summary of equipment, techniques and their accuracy

Routinely, all tumors are considered as metabolically equal regardless of their basal metabolism. In contrast, genetic aberrations are starting to be evaluated as a part of more personalized drug therapies, but their repercussions on metabolism are rarely assessed [188,189]. In our opinion, information about the ability of cancer cells to switch metabolic fuels is fundamental to improve therapeutic success and translational potential of basic research.

**Figure 3:**
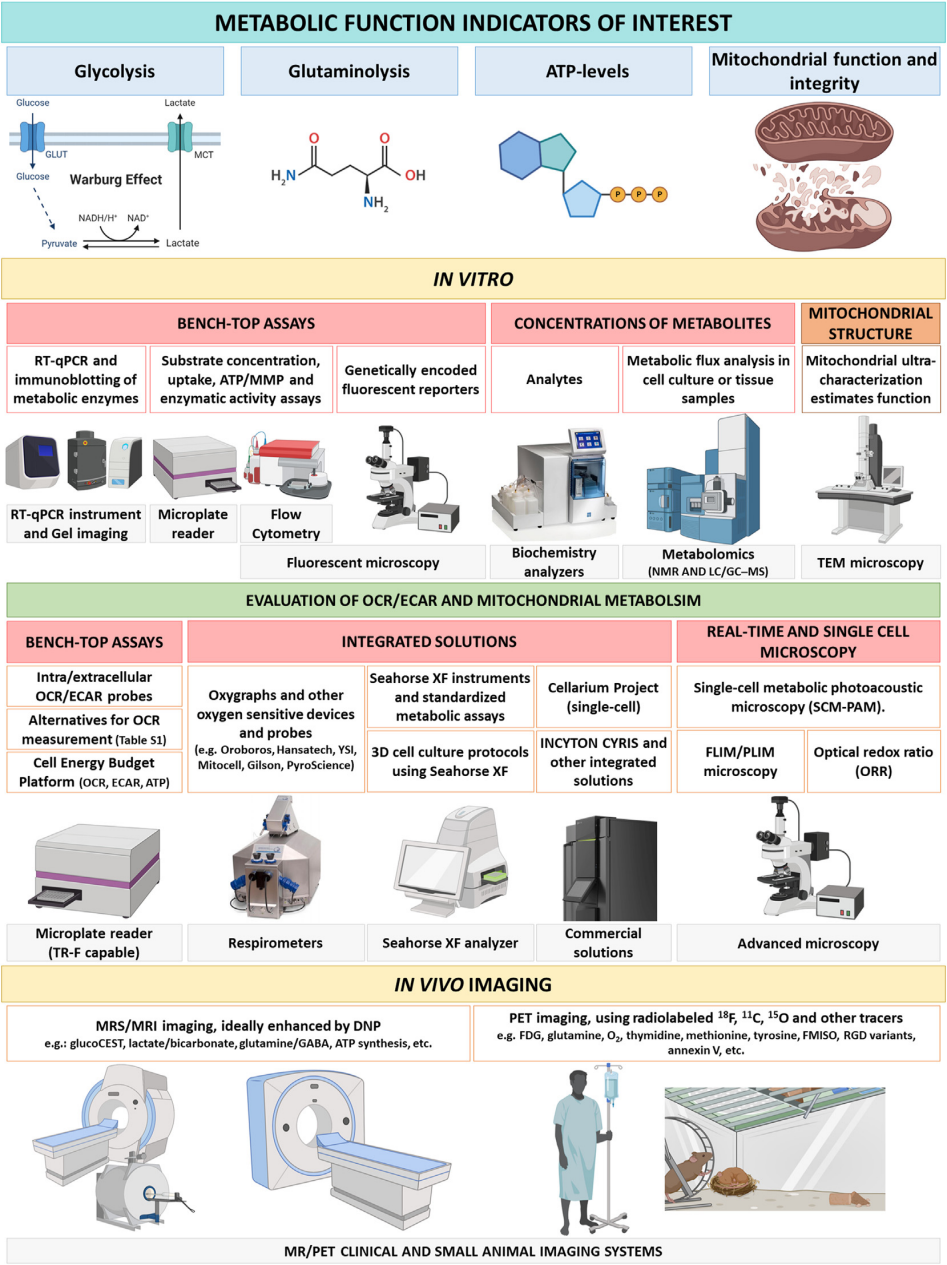
**Methods to measure cancer metabolism *in vitro* and *in vivo*.** Instruments and platforms required are indicated below each metabolic parameter. Detailed information about individual techniques, protocols and further reading is provided in Table S1.

A summary of techniques to study metabolism is presented in Figure 3 and Table S1. Techniques were separated into *in vitro* and *in vivo* analysis of glycolysis, glutaminolysis, ATP production and mitochondrial function (including OCR, membrane potential, as well as structure and cristae shape). In addition, we propose a simple flow chart clinical model where results from metabolic analysis could guide subsequent treatments (Figure S1).

### 5.1 *In vitro* assessment of bioenergetic metabolism

As the bioenergetic profile of individual cells is under dynamic control of various microenvironmental stimuli, such as nutrient availability, oxygen, and acidity [190], numerous attempts have been made to increase the resolution of direct metabolic measurements. Historically, refinements to the Warburg apparatus [191] and Clark electrode chambers [192,193] allowed to measure oxygen consumption in increasingly reduced volumes, but required a high amount of purified mitochondria and removed cellular regulation of metabolism during isolation [194]. Now, these time-consuming techniques are being substituted by multiple-readout integrated solutions, such as oxygen-sensing fluorophores or oxygraphs [195,196]. Quantitative/functional proteomics can reveal the presence and activity of key metabolic enzymes and transporters in tumor tissues and cell cultures. Indirect estimations of metabolic phenotypes can be completed with bulk to single cell metabolic analysis, ranging in precision, price, and technical requirements.

*In vitro*, multiparameter metabolic analysis can be performed by individual assays or integrated solutions. Bench-top commercial solutions for metabolic assessment (nutrient uptake, enzymatic activity, ATP turnover, oxygen/pH/ROS probes, mitochondrial integrity) are available from most research suppliers.

Optical redox ratio (ORR) by two-photon excited fluorescence (TPEF) imaging of NAD(P)H and FAD co-enzymes designates relative changes in global metabolism and single-cell heterogeneity across cancer cells, organoids and animal models [197,198]. It is nondestructive, real-time and label-free, correlating well with both Seahorse flux and kit-based assays of glycolysis [199].

Glycolysis determination was previously reviewed by Tecla and Teitell, including useful tips and protocols [200]. Overall turnover of glucose, lactate, as well as glutamine and glutamate, can be measured using specialized biochemistry analyzers [201,202]. Bioluminescent assays can be adapted for high-throughput extracellular and intracellular detection of metabolites [203]. Focusing strictly on ATP production, the contribution of glycolysis and OXPHOS can be measured by conventional techniques, including enzymatic methods and targeted luciferase measurements [204]. It should be acknowledged that, even though many manufacturers are competing in this space, most commercial platforms are based on equivalent physicochemical principles (e.g., oxygen sensing probes for determination of OCR, redox-sensitive fluorophores, enzymatic assays). Common limitations include low throughput, single parameter readout, low specificity or signal, susceptibility to changes in pH, careful experimental design, and difficulty for normalization [[205], [206], [207], [208]], Real-time respirometry using extracellular flux of live, adherent cells, is the most widespread technology to measure bioenergetics. In this set-up, mitochondrial respiration is extrapolated from oxygen-sensing electrodes or fluorescent/phosphorescent reporters to determine OCR over time, whereas the Warburg effect is calculated based on extracellular acidification rates (ECAR); specifically, extracellular pH changes due to proton extrusion coupled with lactate, imposing correction by the buffering power of the media and ignoring mitochondrial and pentose phosphate pathway (PPP) contributions to ECAR. OCR and ECAR are the two principal metabolic readouts in this field [209]. Intracellular O_2_ can be compared to extracellular levels as an indicator of single-cell OXPHOS, using phosphorescent Pt-porphyrin-based probes [210,211]. To measure complementary parameters of intracellular metabolism, several exogenous and genetically-induced fluorescent reporters have been developed [61], as detailed in Table S1.

Even though polarography using high-resolution Clark electrodes (e.g., Oroboros oxygraphs) is considered the gold standard for assessing cellular oxygen dynamics, especially when multiplexed with mitochondrial membrane potential (MMP) or ADP–ATP exchange rates [212,213], higher-throughput and user-friendly devices such as Seahorse XF instruments (Agilent) are being validated by a higher number of publications [214]. Oroboros and Seahorse XF are the leading platforms that are still continuously updated [215,216]. Rapid analysis of theoretical glycolytic and oxidative fluxes can be performed using the Seahorse XF and other microplate-based techniques, measuring relative changes in OCR/ECAR in response to different substrates (glucose, glutamine, fatty acids) and metabolic inhibitors [217]. For lower budgets, Takahashi et al. devised an inexpensive alternative for manually measuring OCR of adherent cells in a custom glassware sealed chamber, using an optical oxygen probe [218]. Basic overview of OCR measurements and guidelines have been extensively reviewed elsewhere [61,219,220]. If researchers opt to use extracellular flux analysis, raw OCR/ECAR data can be partitioned using special indexes [221]. Predictive models based on flux analysis have been postulated to quantify bioenergetic health, such as the “Bioenergetic Health Index” for peripheral blood cells [222] and “Mitochondrial Oncobioenergetic Index” for cultured tumoral cells [223].

Unfortunately, extracellular OCR has important caveats with regards to its interpretation as a marker of mitochondrial function. While extracellular flux analysis has become an accepted standard to measure metabolic parameters, without specific substrate inhibition it cannot granularly allocate their contribution to global OCR, be it destined for ATP generation, uncoupled respiration, and other pathways [220,[224], [225], [226]]. Careful attention needs to be devoted to “non-mitochondrial OCR”, that is, cellular processes that consume oxygen outside the mitochondria, which has been shown to account for a large portion of OCR [194,227]. Contrary to what would be expected, data from numerous triple fuel inhibition Seahorse experiments (simultaneous targeting of pyruvate, glutamine, and FAO, detailed in Table S3) achieved only a small reduction in total OCR, suggestive of dysfunctional mitochondria or resistance to pathway inhibitors. Caution is advised when interpreting Seahorse results, as relative changes of basal OCR under mitochondrial inhibition are commonly presented as functional OXPHOS, but, OCR cannot fully evaluate mitochondrial function [29,[228], [229], [230]]. Schmidt et al. expertly reviewed the methodological concerns and limitations of extracellular flux analysis, recapitulating the high variability in coupling efficiency, need for normalization, avoidance of non-physiological and artefactual conditions, while also providing a workflow to reduce ambiguity when interrogating mitochondrial function [231]. OCR/ECAR readings should be complemented using additional techniques such as force flow analysis and single cell imaging to interrogate ATP synthesis, MMP, and confirm mitochondrial integrity.

For this purpose, Papkovsky et al. proposed a comprehensive protocol for bioenergetic assessment, the “Cell Energy Budget Platform” (CEB) [232]. Contrary to the Seahorse XF, CEB is designed to measure an extensive set of bioenergetic parameters without specialized equipment, except a time-resolved fluorescence reader. The CEB uses commercial fluorescent or luminescent probes and can be setup in a multi-well format to measure OCR, ECAR/pH (lactate and lactate/CO_2_ extrusion), ATP production and total protein for normalization, as well as MMP, mitochondrial pH, redox state, NAD(P)H, and Ca2+.

An affordable and simple alternative for the measurement of oxygen uptake in biological samples has been developed using the Redflash technology from PyroScience [233]. This device can be adjusted to determine cell respiration, lactate excretion, and MMP where measuring actual lactate excretion is more specific of glycolysis than ECAR [234]. An added advantage is that oxygen-sensing optodes can be reutilized for years, circumventing repeated purchase of single-use cartridges [235].

The Cellarium platform was presented as an integrated solution with a significantly higher resolution, allowing for single cell analysis and confirming differences in the metabolic profiles of nearby cell populations [236]. Following these innovations, Hai et al. developed a high-throughput platform to assess OCR using single-cell metabolic photoacoustic microscopy (SCM-PAM), based on the hemoglobin exchange of oxygen in microwell arrays [237]. This method also avoids bulk-averaged measurements and further demonstrated metabolic heterogeneity at the cellular level. To match these technological advances, Agilent recently launched the Seahorse XF HS instrument with improved signal ratios and sensitivity [216].

Obtaining preclinical models to study tumor heterogeneity without the need of repeated, multi-site biopsies, is being facilitated by isolating circulating tumoral cells [238,239]. Still, the analysis of metabolism at the single-cell level is laborious and costly, not only by the intrinsic difficulty of isolating single cells, but also due to challenging computational workflows; therefore, streamlining these developments will be necessary to widen its adoption [240,241]

Single-cell analysis of intercellular heterogeneity should be examined in light of the tumoral microenvironment. Maintaining 3D cell-to-cell interactions and tissue structure could be crucial to truly recapitulate metabolic heterogeneity and avoid confounding factors of 2D cultures. Seahorse technology was adapted into a workflow to study 3D cultures and complex microtissues (organoids/organotypic models) [242]. Direct metabolic examination of tumor biopsies and healthy tissues is bridging the gap between *in vitro* and clinical studies [243,244]. Recently, a methodological summary for the characterization of organoids using the Seahorse technology was presented, circumventing common pitfalls by DNA-based normalization [245].

Statistical analysis of OCR/ECAR is especially problematic with a low number of samples, requiring careful design to avoid intra and inter-experimental background noise, invalid measurements, and outliers. Raw Seahorse XF measurements carry a complex data structure that requires advanced statistical modeling (e.g., OCR-Stats, OCRbayes) [219,246].

We consider the lack of absolute normalization as the main limiting factor to compare OCR/ECAR measurements between research groups. Currently, there is no standardized set of methods for normalization, leaving each researcher to normalize their experiments as they see fit. Data are regularly presented without advanced normalization, assuming it is comparable as long as an equal number of cells was seeded into each well, or normalized *ad-hoc* to protein/DNA content or cell number for each batch of experiments [247]. Therefore, inter-laboratory setups are personalized and virtually incomparable. As metabolism is highly dynamic due to cell handling and experimental conditions, as well as adjusted to specific normalization factors, the same cell lines measured in different circumstances can deliver vastly divergent results [[248], [249], [250], [251]]. Flux rates, adjusted to cell number or DNA/protein, can be representative within the same experimental setup, but do not translate between research groups [247]. There have been strides towards a more universal normalization crystalized in Agilent's Seahorse XF Imaging and Normalization system, estimating cell number from brightfield and fluorescence images acquired by a compatible BioTek Cytation 1/5 Cytometer [252]. However, this upgrade is yet to see wide adoption and requires a separate instrument purchase. Complete platform integration would offer a standardized normalization factor across all applications.

**Figure 4:**
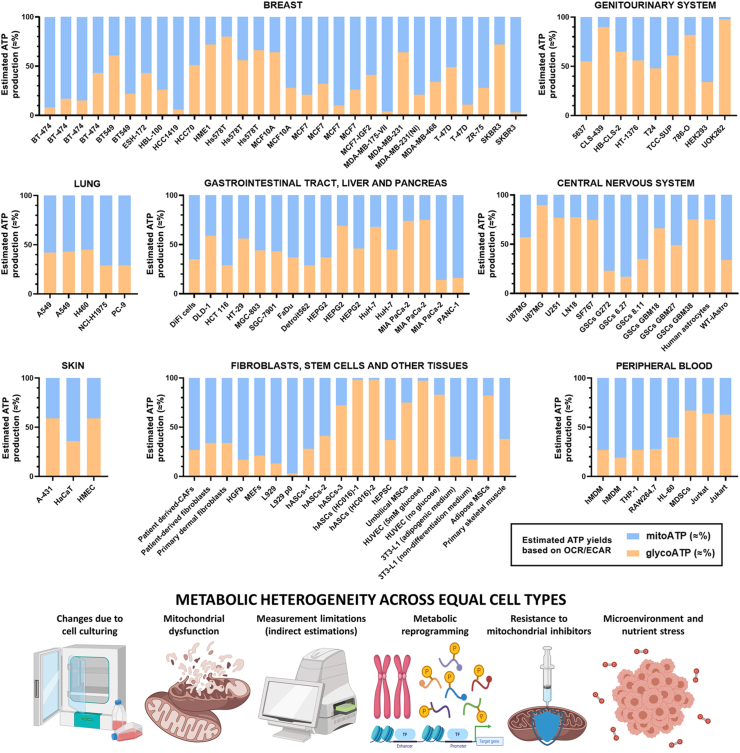
**ECAR-linked (glycoATP) and OCR-linked (mitoATP) energy production, expressed as percentage of total ATP determined by Seahorse XF Real-Time ATP assays, in different cell lines and tissue origins.** The ATP values are based on general stoichiometries, not cancer specific OXPHOS metabolic fluxes, and thus they represent only an assumed mathematical extrapolation. ATP is not being determined experimentally in this assay. High metabolic variability should be noted even in the same cell subtypes, especially when measured by independent authors (e.g., breast cancer cell lines SKBR3, BT549, Hs578T and T-47D; pancreatic cell line MIA PaCa-2; hepatic HEPG2 and others). Numerical values, methodology of analysis and references are provided in Tables S2 and S3.

A nondescriptive library of results acquired using Seahorse XF technology is assembled in the Cell Analysis Database from Agilent, but no attempts have been made to integrate this information. For the first time, we recapitulate metabolic heterogeneity *in vitro* determined by Seahorse XF technology in Figure 4. Seahorse Real-Time ATP assays (Figure 4 and Table S2) and Mito Fuel/Substrate Oxidation assays (Table S3) were selected for direct comparison between cell lines, irrespective of internal normalization factors. Raw OCR and ECAR rates cannot be accurately compared in different experimental setups unless following the same methodology for normalization. It is important to note that relative changes in OCR/ECAR-linked ATP ignore absolute ATP production rates, which can differ greatly depending on the basal metabolic activity of the cells.

In conclusion, mitochondrial dysfunction and the two-compartment model of cancer described earlier present numerous open questions that metabolic studies at the cellular and whole-tissue level could resolve. Primarily, characterize if oxidative and glycolytic phenotypes in cancers with proven mitochondrial defects are strict metabolic categories or various degrees of plasticity. Secondly, characterize if adaptive capacity evolves as a function of disease progression. Regarding metabolic therapy, glycolytic and oxidative compartments should be determined and tackled simultaneously to improve therapeutic efficacy.

### 5.2 Additional considerations about *in vitro* experimental conditions and metabolic assays

Artificial environments need to be considered as powerful determinants of metabolic regulation. Metabolic changes can be partially owed to chemically treated, immortalized cell lines; arguably, such cells do not fully retain original metabolic phenotypes due to changes in nutrient concentrations, growth factors, lactate, oxygen/pH ranges, loss of complex 3D interactions, and other factors [[253], [254], [255]]. Cell culturing is directly responsible for several characteristics of altered metabolism, especially the interplay between the Warburg and Crabtree effect (an artifact of *in vitro* studies where fermentation represses respiration even under hyperoxia) [256]. Transformation is triggered by adherent cell growth and repeated cell detachment (metastatic phenotypes) [[257], [258], [259]], with cells having to transiently adapt to unfavorable medium compositions, as shown by glucose/glutamine deprivation experiments [260,261]. Mitochondrial defects can arise by subculturing, further obscuring oxidative ATP production [48]. Differences in cell handling and microenvironmental factors such as hypoxia can significantly alter readouts of direct measurements of metabolism in cell culture [66,262]. *In vivo*, nonlinear microenvironmental gradients regulate the metabolic state of tumoral cells [263,264]. Ideally, metabolic analysis would be performed on fresh tumor samples and organotypic/organoid models [265,266], or with the lowest passage number [267]. In recent years, significant strides have been made to design more physiological cell culture media and to characterize the artifacts induced by an abnormal *in vitro* microenvironment [[268], [269], [270]]. Recognizing this issue and contrasting metabolic measurements performed in traditional cell culture with more representative and nuanced models could help to bridge the translational gap of metabolic therapy.

### 5.3 Metabolic imaging (PET/MRS)

Metabolic imaging is a noninvasive approach to measure bioenergetics *in vivo*. Positron emission tomography (PET) and proton magnetic resonance spectroscopy (^1^H-MRS) are the two most widely adopted techniques. PET allows to visualize radioactive-labeled molecules as they travel across the body, with simultaneous anatomic localization by computed tomography (CT) or magnetic resonance imaging (MRI). Similarly, MRS is an MRI-based technique for the detection of spectral bands corresponding to several metabolites without the need for ionizing radiation. Acquisition can be performed in a relatively short time (10–15 min) but, without signal enhancements such as hyperpolarization, it is limited by low sensitivity.

To estimate glycolysis *in vivo*, PET-imaging with glucose analogues such as ^18^F-fluorodeoxyglucose (^18F^-FDG) is used to identify areas of high glucose uptake (GLUT1/3). ^18^F-FDG has been standardized to image most anatomical enclaves. Despite poor sensitivity for tumors with low glycolysis and substantial cerebral/inflammatory background, FDG-PET has become an essential clinical tool for staging and response assessment [271,272]. To complete substrate allocation, alternative PET radiotracers are being investigated, as summarized in Table S1. One important limitation is that PET imaging can only evaluate uptake of tracers, which does not always correlate with real processing for fuel or biosynthesis.

MRS, in conjunction with metabolomic profiling, are a helpful combination to study intracellular flow of metabolites without radiolabeled tracers. ^7^O-MRS and ^31^P-MRS enable estimation of oxygen consumption and mitochondrial ATP synthesis, whereas ^9^F-MRS has been proposed for measurement of glycolysis [273]. Metabolite concentrations correlated with survival [274,275], with quantification of metabolites such as glutamine, TCA cycle intermediaries, and lipids. Analysis of intratumoral/peritumoral pH and metabolites by enhanced ^1^H-MRS methodologies such as the “chemical exchange saturation transfer” (CEST) and “biosensor imaging of redundant deviation in shifts” (BIRDS) allow for precise evaluation of glycolysis [276,277]. CEST can be used to detect the signal of glucose (glucoCEST) and lactate. T1ρ-weighted dynamic glucose-enhanced (DGE)-MRI has been used to reveal *in vivo* glucose metabolism [278]. d-glucose is also being evaluated as a biodegradable contrast, but its use to monitor metabolic responses remains unclear [279].

Under this section, we would like to highlight the promise of novel dynamic nuclear polarization (DNP)-enhanced ^13^C tracers, which allow to study pyruvate to lactate/bicarbonate flux, KBs uptake, and other pathways such as the PPP or redox capacity, with improved signal ratios [280,281]. Integration into routine clinical practice could significantly improve pre/post-treatment metabolic evaluation [282].

Unfortunately, while conventional PET/MRS imaging can provide an overview of preferences towards specific metabolites, it does not inform about distribution inside cells. At this time, a wider array of methods should be employed to obtain a complete metabolic picture. There have been some successful pilot studies that included combined metabolic measurements; for example, Neveu et al. determined FDG uptake, lactate production, tumor oxygenation, and metabolic fluxes *in vivo*, using PET, microdialysis, electron paramagnetic resonance imaging, and [^13^C]-hyperpolarized MRS, respectively [283,284]. CTs and case reports evaluating the safety and efficacy of metabolic therapies often include some form of basic metabolic imaging (mostly FDG-PET), but rarely direct tissue-level metabolic analysis, so patient stratification remains an unresolved problem [187,285]. In clinical contexts, reduced access to precise and easy-to-use tools to measure relevant metabolic information is likely responsible for this limitation.

### 5.4 Metabolomics and metabolic profiling

Metabolic profiling, using metabolomics, is the measurement of low-molecular-weight metabolites and intermediates from biological samples, and offers a panoramic view of the dynamic response to molecular and pathophysiological stimuli [[286], [287], [288], [289]].

Flux of metabolites through metabolic pathways is key to understand how cancer cells meet their bioenergetic demands. A wide variety of metabolomic techniques can identify cancer related metabolites, such as liquid and gas chromatography-mass spectrometry (LC/GC–MS) and nuclear magnetic resonance spectroscopy (NMR). Dynamic characterization of metabolism is a daunting task due to cellular adjustments to everchanging concentrations and microenvironments [290,291]. Stable isotope-resolved metabolomics (SIRM) is a tool for such dynamic studies, illustrating the mechanism of action of pathway inhibitors and quantifying downstream metabolites [292,293].

Raw metabolomic data can be processed by “Pathway Activity Profiling (PAPi) to predict dysregulated metabolic pathways [294]. MS-based metabolomics are particularly relevant for direct evaluation of bioenergetic metabolism in bulk biological samples [295]. Clinical data from a broad range of cancers suggests that metabolomics can improve classification of biopsies [286]. In conclusion, when available, metabolomic approaches could be applied for patient stratification, especially as a first order overview of biopsies. Accurate tracing of metabolites uncovers a more detailed picture of their distribution than averaged OCR/ECAR measurements [296,297].

### 5.5 Mitochondrial structure and mtDNA mutations as indicators of mitochondrial function

Mitochondria are bioenergetic, biosynthetic and signaling organelles, playing a vital role in adaptation to changes in the microenvironment and tumorigenesis [298]. Ultrastructural characterization of mitochondrial membranes provides insights about the relative state of the mitochondria in a pool of cells, but it is not easy to perform and requires specialized equipment [299]. As previously discussed, the most widespread tool to assess mitochondrial function indirectly is to evaluate OCR fluctuations under sequential ETC inhibition. Nevertheless, mitochondrial health in cancer is a source of controversy. To examine function in light of structure, an argument has been made for the identification of structural defects, mitochondrial mass, and mtDNA mutations [29]. As cytotoxic drugs have been shown to inflict structural damage to the mitochondria, and resistance to chemotherapy is linked with their dysfunction, analyzing mitochondrial fitness may be warranted in chemoresistant tumors [[300], [301], [302]].

Moreover, mtDNA mutations have been proposed as biomarkers for early detection, acting as negative regulators of OXPHOS [[303], [304], [305]]. In certain cancers, such as bladder, lung, and head/neck tumors, mtDNA mutations were even more abundant than nuclear mutations [306]. Increased mitochondrial biogenesis is essential for tumorigenesis [307], and analysis of total mitochondrial mass reveals differences between tumoral and normal cells [308,309]. Although the functional effects of all mtDNA mutations have not been fully elucidated [310,311], mitochondrial intercellular transfer and transplantation experiments hold the promise of restoring oxidative function, reversing cancer proliferation and overcoming therapeutic resistance [[312], [313], [314], [315]].

## 6 Concluding remarks

For appropriate metabolic therapy, substrate flexibility needs to be studied in advance, as cancer subtypes will show distinct fuel preferences. To achieve this, we summarized techniques to measure key metabolic parameters *in vitro* and *in vivo*, accommodating different research needs and budgets. Our straightforward flow chart could be used to approach clinical metabolic analysis. Taking all these complexities into account will advance our understanding of the contribution of metabolism to cancer origin and progression. Where characterization drives subsequent treatment, the collection of metabolic data in different tumor specimens, interventions and microenvironments is essential. Our hope is that, as more researchers contemplate including metabolic measurements in their studies, bioenergetic classification will unlock fundamental insights for improved cancer treatments.

## Author contributions

Writing - original draft, T.D.; Writing - review & editing, T.D., N.G.-R., T.N.S.; Visualization: P.C.N.; Supervision: A.A.-S. All authors have read and agreed to the published version of the manuscript.

## Funding

This research was funded by grants from the “Fondo de Investigaciones Sanitarias” (FIS) (PI17-01489), the 10.13039/501100003329Ministerio de Economía y Competitividad (RTC2019-006918-1). Research funded by 10.13039/501100003176Ministerio de Educación, Cultura y Deporte (FPU16/03198).

## Data availability statement

The data presented in this review are available and individually cited in the Supplementary Materials.
