# Supplementary material for: Metabolic therapy and bioenergetic analysis: The missing piece of the puzzle
Source: Mol Metab. 2021 Nov 5;54:101389. doi: 10.1016/j.molmet.2021.101389 (PMC8637646; doi:10.1016/j.molmet.2021.101389)
Supplement: Multimedia component 1 [file mmc1.pdf]

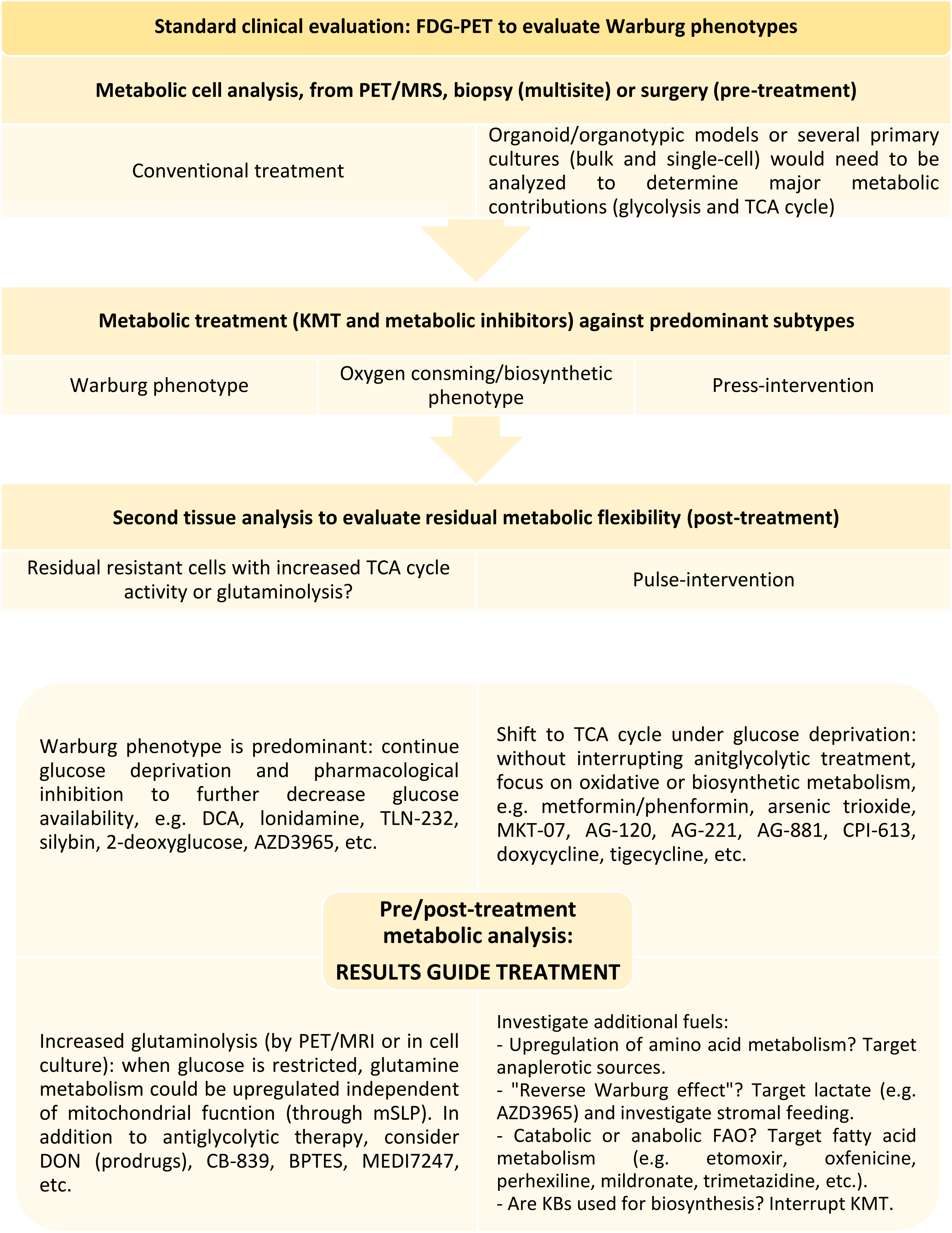

Figure S1. Adjustable flow-chart for clinical metabolic analysis. FDG-PET can identify high glucose uptake *in vivo*, to be confirmed by analyzing tumoral tissue at the cellular level. Warburg-like phenotypes should be addressed first via anti-glycolytic therapy. If other predominant metabolic subtypes are detected during or after standard of care and/or metabolic therapy, they would need to be addressed separately using specific interventions.
