# Supplementary material for: Metabolic therapy and bioenergetic analysis: The missing piece of the puzzle
Source: Mol Metab. 2021 Nov 5;54:101389. doi: 10.1016/j.molmet.2021.101389 (PMC8637646; doi:10.1016/j.molmet.2021.101389)
Supplement: Multimedia component 2 [file mmc2.pdf]

**Method to measure metabolism**	**Technical details and examples**	**Readouts**	**Instruments required**
Antibodies against glycolytic enzymes, e.g.:<br><br>GLUT transporters, HK1/2, PFK, PFKFB1-4, Aldolase, PGAM1, Enolase 1-3, GAPDH, PKM1/2, LDH, PDH complex, PDK, and others.	Western Blotting and immuno-based techniques. Gene and protein expression patterns under the specific experimental conditions can give a preliminary idea of averaged metabolic states [1-5].	Presence/absence and phosphorylation status of enzymes.	Detection imaging system and basic research instrumentation (immunoblotting).
Glycolytic pathway enzymatic activity assays.	Most research suppliers offer standard enzymatic assays. Examples:<br>LDH, *Abcam, ab102526; Biovision, K726; Sigma, MAK066 (OD 450nm); Promega, J2380 (luminiscence); BioLegend, 426401 (A490 nm).* PDH, *Abcam, ab109902; Biovision, K679; Sigma, MAK183 (OD 450nm).* PK *Abcam, ab83432; Biovision, K709; Sigma, MAK072 (OD 570 nm and Ex/Em = 535/587 nm).* G6P, *Abcam, ab83426; Biovision K657; Sigma MAK014 (OD 450nm).* G6PDH *Abcam, ab176722 Cayman 700300; Sigma, MAK015 (OD 450nm or Ex/Em = 540/590 nm).*	Relative enzymatic activity.	Microplate reader (colorimetric, fluorometric, luminescence).
qRT-PCR for relative expression of glycolytic, glutaminolytic and OXPHOS enzymes.	Standard protocol and considerations about transcriptional metabolic reprogramming at [6; 7]. Most research suppliers offer validated primers for metabolic enzymes.	Relative comparison of gene expression (mRNA) in cell culture.	Real-Time qRT-PCR instruments (common vendors, e.g. Agilent, Bio-Rad, Thermo Fisher, Qiagen, etc.).
In vitro 2-NBDG uptake.	General protocol at [8]. Kit-based, e.g., *Abcam, ab235976; Biovision K682; MyBioSource MBS846815; ATT Bioquest 23500; Cayman 600470; LSBio, LS-K620. Readout by flow cytometry (488 nm) or fluorescence (excitation range 420 nm-495 nm).*	Glucose uptake.	Microplate reader (colorimetric, fluorometric, luminescence).<br><br>Flow cytometry.<br><br>Fluorescence microscopy.
In vitro 2-DG uptake.	Kit- based solutions: e.g., *Abnova KA3751 (Ex/Em = 535/587 nm); Sigma MAK083 (OD 412nm); MAK084 (Ex/Em = 535/587 nm); LSBio K618 (OD 412 nm); Promega J1341 (luminescence); Promocell PK-CA577-K666 (Ex/Em = 535/587 nm).*	Glucose uptake.	Scintillation counter (radioactive, uncommon).<br><br>Microplate reader (colorimetric, fluorometric, luminescence). Fluorescent microscopy.
In vitro ^{18}FDG uptake.	Uncommon method due to radiation hazard.	Glucose uptake. Radiation (no commercial kits)	Scintillation counter/cyclotron.

Glucose reserves (intracellular).	Protocol at [9] and additional measurements (such as pH/ROS) reviewed at [10]. Allows for repeated measurements, non-destructive.	Change in pH and impedance due to glucose oxidase interaction with glucose. Direct correlation with single cell intracellular glucose levels.	Custom intracellular glucose nanopipettes.
Antibodies against TCA cycle enzymes, e.g.:<br>SUCLG1/2, MPC1/22, Fumarase, IDH1/2, Citrate Synthase, ACO1/2, MDH 1/2, ACLY, DLST, PDH complex, PC, PCK1/2, SDHA/B/C (complex II)	Western Blotting and immuno-based techniques	Presence/absence and phosphorylation status of enzymes.	Detection imaging system and basic research instrumentation (immunoblotting).
Antibodies against ETC, e.g.:<br>SDHA/B/C (complex II), Complex I, Complex III (NDUFS4, NDUFB3), Complex IV (cytochromes), ATP Synthase, ETFDH, WDR93.	Western Blotting and immuno-based techniques.	Presence/absence and phosphorylation status of enzymes.	Detection imaging system and basic research instrumentation (immunoblotting).
TCA cycle and ETC enzymatic activity.	Most research suppliers offer standard enzymatic assays. Examples:<br>*CS,* e.g., *Abcam, ab119692; Sigma, MAK193; Biovision K318 (OD 412 nm)*<br>*ETC I, Sigma; MAK359 (A600 nm); ETC III, Sigma; MAK360 (A550 nm); ETC V, Abcam; ab109716 (OD 340 nm);*<br>*SDH, Sigma, MAK197; Abcam, ab228560; Biovision, K660 (OD 600 nm);*<br>*SUCL, Sigma, MAK217; Abcam, ab196989; Biovision K597 (OD 450 nm).*	Relative enzymatic activity.	Microplate reader (colorimetric, fluorometric, luminescence).
Antibodies against glutaminolytic enzymes, e.g.:<br>Mainly GLS1/2, GLUD1/2, GS, GPT1/2, GOT1/2, ALT/AST. Other enzymes such as SUCL (mSLP), KGDHC, NME and DOHDH could be evaluated simultaneously.	Western Blotting and immuno-based techniques.	Presence/absence and phosphorylation status of enzymes.	Detection imaging system and basic research instrumentation (immunoblotting).

Glutaminolysis enzymatic function assays (GLS, GLUL, GS, GDH, etc.).	Most research suppliers offer standard enzymatic assays. Examples: GLS, *Abnova, KA1627 (OD 565 nm); Biovision, K455 and K479 (Ex/Em = 535/587 nm); MyBioSource, MBS8243221 (OD 420 nm); Promocell, PK-CA577-K45 (Ex/Em= 535/587); BMR, E-133 (OD 492 nm); MyBioSource, MBS8243221 (OD 420 nm).*	Enzymatic activity.	Microplate reader (colorimetric, fluorometric, luminescence).
Glutamine and glutamate kit-based measurement. Changes in glucose/lactate and other metabolites can also be determined using bench-top kits.	Most research suppliers offer standard substrate determination assays. Examples: *Abcam, ab197011 (OD 450 nm); Abnova, KA1627 (OD 565 nm); Abnova, KA4553 (0D450); Biovision, K556-100 (OD 450 nm); MyBioSource, MBS841633 (OD 450 nm); Promega, J8022 (luminescence); Sigma, GLN1-1KT (OD 339 nm).*	Changes in concentration of glutamine (extra/intracellular).	Microplate reader (colorimetric, fluorometric, luminescence).
Fatty acid metabolism kit-based assays.	Examples: Fatty Acid Uptake*: Sigma, MAK156-1KT; Ex/Em = 485/515 nm; Abcam, ab176768 (Ex/Em = 485/515 nm); QBT R6132; Promocell PK-CA577-K408 (Ex/Em = 488/523 nm) QBT, R6132 (Ex/Em = 485 /515nm); Biovision, K408 (Ex/Em: 488/523 nm)* β-Hydroxybutyrate Assays*: Sigma, MAK041-1KT, (OD 570 nm); Sigma, MAK134-1KT; OD 340 nm; Abcam, ab272541 (OD 340nm); EnzyChrom EKBD-100 (OD340nm); Cell Biolabs MET-5082 (OD 450 nm); Cayman, 700190 (A450).* Ketolysis might be an advantageous metabolic state for tumoral cells, as upregulation of ketolytic enzymes and KBs is a trait of some aggressive forms of cancer [11-17].	Fatty acid and derived molecules transport and levels.	Microplate reader (colorimetric, fluorometric, luminescence).<br>Fluorescent microscopy.
Overall turnover of metabolites using specialized biochemistry analyzers.	Commercial devices are usually based on the enzyme-sensor technology from YSI (e.g., YSI 2300/YSI 2700/YSI 2900). Standard protocol for the YSI 2900 at [18]. Alternatives to YSI are reviewed at [19].	Enzyme-sensor technology for the analysis of multiple metabolites, such as glucose, glutamine, glutamate, lactate, ammonium, and others.	Biochemistry analyzers.
ATP and ATP/ADP levels and ATP-based viability assays.	Conventional methods reviewed at [20]. Most research suppliers offer ATP kits. Examples: *Abcam, ab65313 (luminescence); Abcam, ab83355 (Ex/Em = 535/587 nm, OD 570 nm); Abcam, 113849; (luminescence); Cayman Chemical, 700410 (luminescence); Enzo, ALX-850-248 (luminescence); Enzo; ALX-850-247 (luminescence); Invitrogen, A22066; Ex/Em = NA/560; Sigma, MAK190-1KT (Ex/Em = 535/587 nm, OD 570 nm).*	ATP levels, ATP/ADP ratio.	Microplate reader (colorimetric, fluorometric, luminescence).<br>Flow cytometry.<br>Fluorescence microscopy.
ATP detection using HPLC combined with fluorescence detection.	Protocols available [21; 22].	ATP levels.	HPLC instruments (e.g. Agilent, GE Healthcare, Biobase, Hitachi, PerkinElmer, etc.)
Mitochondrial Membrane Potential assays, usually using JC (JC-1/JC-10),	Most research suppliers offer MMP kits. Examples: JC-1 based: e.g., *Abcam ab113850; Cayman 10009172; Elabscience-E-CK-A301; Thermo*	MMP collapse and changes in MMP.	Microplate reader (colorimetric,

TMRE, MitoID, DiOC2(3) and MitoTracker-based probes.	*M34152 and others; Ex/Em = 540/570nm and Ex/Em = 485/535nm.* JC-10 based: e.g., *Sigma, MAK159-1KT and MAK160-1KT; Abcam ab112134; Enzo ENZ-52305 and others; Ex/Em = 540/590nm and Ex/Em = 490/525nm.* Other probes: *Abcam, ab113852 (Ex/Em 549/575 nm); Cell Signaling-13296S (Ex/Em = 550/580 nm); Enzo; ENZ-51018 (Ex = 488nm); Sigma, MAK146 (Em = 535 nm); Sigma, MAK147 (Ex/Em = 540/590nm); Sigma, MAK149 (Ex/Em = 640/680nm); Thermo M34150 (Ex/Em = 482/497 nm); Thermo MitoTracker (V35116, M7510, M7512, M7514) (variable fluorescence).*		fluorometric, luminescence). Flow cytometry. Fluorescence microscopy. Variable Ex/Em depending on assay (approximate range 480/680 nm).
Live cell imaging using fluorescent reporters.	Reviewed at [23]. Reporters include, e.g. 2-deoxyglucose analogs [24], or genetically induced intra-cellular and intra-mitochondrial pH sensors (*pHRed, pH-Lemon*) [25; 26], FRET-based glucose, pyruvate and glutamine nanosensors [27-29], ATP sensors (*Perceval, mitoATeam analogues)* [30], MMP via pH-sensitive yellow fluorescent protein (*mitoSypHer, mt-EYFP analogues, mtAlpHi*) [31], hypoxia [32; 33], Ca^{2+} and ROS reporters [34] and NADH:NAD+ ratio biosensors (*Peredox*) [35]. As hypoxia plays a central role in the fate of pyruvate, pimonidazole hydrochloride (*Hypoxyprobe*) identifies hypoxic regions within a tumor using immunohistochemistry and *in vivo* protocols [36].	Genetically encoded fluorescent reporters to evaluate intracellular metabolism.	Fluorescence microscopy.
Bench-top intra/extracellular oxygen consumption/uptake assays.	Multiple manufacturers; usually based on the MitoXpress probe or equivalent physicochemical principles. Examples (extracellular): *Abcam ab197243, or dye only, Abcam, ab197242 (Ex/Em = 360-380 nm /630-680 nm); Agilent, MX-200-4 (Ex/Em = 340/642 nm); Cayman Chemical, 600800 (Ex/Em = 380/650 nm); Cayman 601060 (dual OCR/ECAR; Ex/Em = 380/650nm and A490 nm); Enzo, ENZ-51045 (Ex/Em = 340-380/630-680 nm).* Examples (intracellular): *Abcam, ab197245 (Ex/Em = 340/642 nm); Agilent, MX-300-4 (Ex/Em = 380/645 nm); Enzo, ENZ-51046 (Ex/Em =380/650nm).*	Intra/extracellular OCR.	Microplate reader (fluorometric), preferably with dual-read TR-F (lifetime) capability
Glycolysis assays using soluble pH probes.	Primarily based on the pH-Xtra probe or equivalent physicochemical principles. Examples: *Agilent, PH-200-4 (Ex/Em = 380/615 nm); Abcam, ab197244 (Ex/Em = 380/615 nm); Enzo, ENZ-51048 (Ex/Em = 380/615 nm).*	ECAR.	Microplate reader (fluorometric), preferably with dual-read TR-F (lifetime) capability.
Semi-integrated protocol for OCR/ECAR measurement.	Cell Energy Budget Platform, protocol at [37].	MitoXpress-Xtra (OCR), pH-Xtra (ECAR), total ATP and total protein.	TR-F capable microplate reader.
Inexpensive bench-top protocol to measure OCR.	Protocol for cancer cell culture at [38].	OCR.	Glassware sealed chamber and Ru-solution

			as an optical oxygen probe.
Inexpensive, durable, luminescent oxygen-sensitive sensor.	Protocol for cancer cell culture at [39].	OCR, can be combined with measurement of lactate excretion and MMP.	Redflash technology (PyroScience).
Additional bioluminescent and phosphorescence-based oxygen sensitive probes.	Protocols and descriptions at [40; 41].	Extracellular and intracellular OCR.	TR-F capable microplate reader.
Extracellular flux analysis using Seahorse XF technology.	Availed by the highest number of publications [42]. Raw data can be partitioned using special indexes [43] and should undergo careful statistical analysis (e.g. OCR-Stats, OCRbayes) [44; 45]. Kits available to date, with their respective inhibitors:<br>*-Seahorse XF Real-Time ATP Rate Assay, 103592 (oligomycin, rotenone/antimycin A)*<br>*-Seahorse XF Cell Mito Stress Test, 103015 (oligomycin, FCCP, rotenone/antimycin A)*<br>*-Seahorse XF Palmitate Oxidation (102720) (palmitate:BSA + etomoxir + oligomycin, FCCP, rotenone/antimycin A)*<br>*-Seahorse XF Mito Fuel or Substrate Oxidation, 103260 (etomoxir, UK5099, BPTES + oligomycin, FCCP, rotenone/antimycin A)*<br>*-Seahorse XF Glycolytic Rate, 103344 (rotenone/antimycin A + 2DG)*<br>*-Seahorse XF Glycolysis Stress Test, 103020 (glucose + oligomycin + 2DG)*<br>*Seahorse XF Cell Energy Phenotype, 103325 (oligomycin, FCCP).*	Measures relative changes in OCR/ECAR in cell culture upon pathway inhibition. Can be adapted for isolated mitochondria [46].<br>Normalization is usually performed via cell number, protein or DNA content.	Seahorse XF instruments: XFp (discontinued), HS Mini, XFe24, XFe96.
High-resolution resirometry using OROBOROS Oxygraphs.	Protocol at [47].	OCR	OROBOROS Oxygraph-2k.
Classical polarography using a Clark electrode.	Protocols and descriptions at [48; 49].	OCR (cell culture and isolated mitochondria).	Clark-electrodes or integrated systems (currently active manufacturers, e.g.: Oroboros, Hansatech, YSI, Mitocell, Gilson Medical).
3D cell culture and microtissues, OCR/ECAR measurement based on Seahorse XF technology.	Protocols at [50; 51].	OCR of 3D structures.	Seahorse XF analyzer with adapted plate format and vibratome.

Combined analysis and proprietary integrated solutions.	Cellarium platform described at [52; 53].	OCR/ECAR. Multiplexable using additional sensors (glucose), high-throughput and with time-resolved capabilities.	Special microarray using an oxygen, pH and oxygen/pH insensitive reference probe (Rho-MA).
Combined analysis and proprietary integrated solutions.	CYRIS CORE and CYRIS FLOX platform from INCYTOИ, or 6xIMOLA-IVD cell metabolism monitor from Cellasys.	OCR, ECAR, dissolved oxygen, cellular impedance, microscopic imaging.	Recent launch, pending publications.
Single-cell metabolic photoacoustic microscopy (SCM-PAM).	Protocol at [54]. Purified red blood cells (RBCs) surrounding the sample and single-cell homogenization are required. ECAR would need to be determined prior to addition of RBCs due to their metabolic characteristics [55].	Frequency distribution of single-cell OCR (fmol/min) based upon oxygen saturation of hemoglobin (sO2).	Photoacoustic microscopy
Optical redox ratio (ORR).	Further reading at [56-59].	Relative changes in metabolism based on NAD(P)H and FAD autofluorescence. Good correlation with Seahorse XF.	Two-photon excited fluorescence (TPEF) microscopy.<br>Microplate reader.
Fluorescence lifetime imaging (FLIM) microscopy.	Protocol at [60; 61].	Cellular O2, NAD(P)H and MMP.	FLIM capable high-speed digitizers and microscopes.
O2 phosphorescence lifetime imaging (PLIM) microscopy.	Protocol at [62].	Ex-vivo real-time tissue OCR imaging.	NanO2-IR (Luxcel Biosciences). Timepix3 or similar imaging system.
PET imaging. Principally, using ^{18}F, ^{11}C, ^{15}O, ^{64}Cu, ^{68}Ga and ^{123}I PET tracers.	Examples: *FDG (glycolysis); glutamine (glutaminolysis); oxygen (hypoxia), glutamate (FSPG); choline (diagnostic); thymidine (diagnostic); methionine (prognostic); tyrosine (prognostic); leucine (protein synthesis); DOTATOC (protein synthesis); acetate (oxidation/lipid synthesis); NH_3 (radiation necrosis); FDOPA; thymidine kinase (TK-1); FMISO (hypoxia); RGD variants (angiogenesis); bevacizumab (angiogenesis); synthetic amino acids: AIB, ACPC, FAMP, FACBC; C-SNAT (apoptosis); annexin V (apoptosis); FBnTP (apoptosis).* Several other tracers in active development [63-67].	High sensitivity (1fM), high spatial resolution (0.1-4mm^3), radioactive hazard, high cost of instruments and tracers, non-standardized protocols, might require cyclotron for short-lived compounds.	Small animal PET scanners for animal models [commercial solutions listed in [68]], combined with CT/SPECT imaging. For clinical trials, PET-CT scanners leading manufacturers, e.g. Siemens, GE, Philips, Mediso, Elysia-raytest).
MRS/MRI imaging. Principally, using ^{1}H, ^{13}C, ^{23}Na, ^{31}P chemical shifts and dynamic nuclear polarization.	Small animal imaging protocol at [69]. *Examples: pyruvate, glucose (glucoCEST), lactate (glycolysis) and bicarbonate, succinate, glutamine/GABA (glutaminolysis, neurotransmitters), ATP synthesis (e.g., ^{31}P ST; EBIT), α-ketoglutarate (TCA cycle), acetate (fatty acid transport), NAAA (diagnostic), lipids, creatine/phosphocreatine (energy*	Need of hyperpolarization to improve sensitivity and signal (0.1-1mM), medium spatial resolution (0.25-6cm^3), no	Preclinical and clinical MRI imaging systems (leading manufacturers, e.g. Siemens, GE, Philips, Hitachi, Toshiba, have available solutions

	metabolism), choline (diagnostic), myo-inositol (diagnostic), alanine, other amino acids. Choline and citrate have been measured primarily in prostate cancer, whereas glutamate/glutamine, glycine, GABA, NAA, lipids, choline, 2HG and succinate are typically investigated in brain and other regions [70-74].	radiation, very expensive instruments, complex application and interpretation.	for both veterinary and clinical scanners).
Metabolic flux analysis using specific NMR and LC/GC–MS techniques.	LC/GC-MS and NMR metabolomics reviewed at [75]. NMR is characterized by low sensitivity and resolution but high reproducibility and more accurate identification, using ^{13}C chemical shifts and labeled metabolites (glucose, amino acids, TCA cycle intermediaries, fatty acids). HPLC-MS, ultra-HPLC (UHPLC), SFC-MS and CE-MS have a wide range of sensitivity, reproducibility, and resolution, but metabolites are difficult to identify due to non-standardized databases. GC-MS is very sensitive, metabolites are easy to identify by spectral libraries, but it requires higher amounts of sample. Relative rates of glucose and glutamine consumption can be determined to improve patient stratification [76-82]	Depending on instrument resolution (ability to distinguish compounds), untargeted and targeted metabolomics.	Spectroscopic and spectrometric instruments, such as high-field NMR and MS (typically performed with quadrupole TOF or ion-mobility mass spectrometers), as well as separation techniques with detection (HPLC, GC, CE, 2D chromatography and supercritical fluid. chromatography).
Mitochondrial ultra-characterization.	Protocols and further reading at [83-86].	Mitochondrial cristae shape informs about OXPHOS function.	High resolution and TEM microscopy.

Table S1. Summary of methods and techniques to study cellular bioenergetic metabolism. Non-standard abbreviations: TR-F (time-resolved fluorescence), MMP (mitochondrial membrane potential), DOTATOC (90Y-[DOTA]0-Tyr3-octreotide), AIB (2-amino[3-11C]isobutyric acid), ACPC (Aminocyclopentane Carboxylic Acid), FAMP (α-methyl-phenylalanine), FACBC (fluorocyclobutanecarboxylic acid), C-SNAT (Caspase-Sensitive Nano-Aggregation tracer), FBnTP (Fluorobenzyl-triphenylphosphonium), ST (saturation transfer), EBIT (electron beam ion trap), SFC-MS (Supercritical Fluid Chromatography-Mass Spectrometry) and CE-MS (Capillary Electrophoresis– Mass Spectrometry).

References:

[1] Ancey, P.B., Contat, C., Meylan, E.J.T.F.j., 2018. Glucose transporters in cancer–from tumor cells to the tumor microenvironment. 285(16):2926-2943.

[2] Bhutia, Y.D., Ganapathy, V.J.B.e.B.A.-M.C.R., 2016. Glutamine transporters in mammalian cells and their functions in physiology and cancer. 1863(10):2531-2539.

[3] Peng, X., Chen, Z., Farshidfar, F., Xu, X., Lorenzi, P.L., Wang, Y., et al., 2018. Molecular characterization and clinical relevance of metabolic expression subtypes in human cancers. 23(1):255-269. e254.

[4] Board, M., Humm, S., Newsholme, E.J.B.J., 1990. Maximum activities of key enzymes of glycolysis, glutaminolysis, pentose phosphate pathway and tricarboxylic acid cycle in normal, neoplastic and suppressed cells. 265(2):503-509.

[5] Chang, H.T., Olson, L.K., Schwartz, K.A., 2013. Ketolytic and glycolytic enzymatic expression profiles in malignant gliomas: implication for ketogenic diet therapy. Nutrition & Metabolism 10(1):47.

[6] Wong, M.L., Medrano, J.F.J.B., 2005. Real-time PCR for mRNA quantitation. 39(1):75-85.

[7] Hawkins, L.J., Al-Attar, R., Storey, K.B.J.P., 2018. Transcriptional regulation of metabolism in disease: From transcription factors to epigenetics. 6:e5062.

[8] TeSlaa, T., Teitell, M.A.J.M.i.e., 2014. Techniques to monitor glycolysis. 542:91-114.
[9] Nascimento, R.A., Özel, R.E., Mak, W.H., Mulato, M., Singaram, B., Pourmand, N.J.N.l., 2016. Single cell "glucose nanosensor" verifies elevated glucose levels in individual cancer cells. 16(2):1194-1200.
[10] Bulbul, G., Chaves, G., Olivier, J., Ozel, R.E., Pourmand, N.J.C., 2018. Nanopipettes as monitoring probes for the single living cell: State of the art and future directions in molecular biology. 7(6):55.
[11] Saraon, P., Cretu, D., Musrap, N., Karagiannis, G.S., Batruch, I., Drabovich, A.P., et al., 2013. Quantitative proteomics reveals that enzymes of the ketogenic pathway are associated with prostate cancer progression. Molecular & cellular proteomics : MCP 12(6):1589.
[12] Labanca, E., Bizzotto, J., Sanchis, P., Yang, J., Shepherd, P.D., Paez, A., et al., 2021. Prostate cancer castrate resistant progression usage of non-canonical androgen receptor signaling and ketone body fuel.
[13] Cui, W., Luo, W., Zhou, X., Lu, Y., Xu, W., Zhong, S., et al., 2019. Dysregulation of ketone body metabolism is associated with poor prognosis for clear cell renal cell carcinoma patients. 9:1422.
[14] Bonuccelli, G., Tsirigos, A., Whitaker-Menezes, D., Pavlides, S., Pestell, R.G., Chiavarina, B., et al., 2010. Ketones and lactate "fuel" tumor growth and metastasis: Evidence that epithelial cancer cells use oxidative mitochondrial metabolism. Cell Cycle 9(17):3506.
[15] Huang, Li, T., Wang, L., Zhang, L., Yan, R., Li, K., et al., 2016. Hepatocellular carcinoma redirects to ketolysis for progression under nutrition deprivation stress. Cell research 26(10):1112.
[16] Zhang, S., Xie, C.J.L.s., 2017. The role of OXCT1 in the pathogenesis of cancer as a rate-limiting enzyme of ketone body metabolism. 183:110-115.
[17] Grabacka, M., Pierzchalska, M., Dean, M., Reiss, K., 2016. Regulation of Ketone Body Metabolism and the Role of PPARα. International journal of molecular sciences 17(12):2093.
[18] Mitra, S., Molina, J., Mills, G.B., Dennison, J.B., 2015. Characterization of the role Rab25 in energy metabolism and cancer using extracellular flux analysis and material balance. Rab GTPases. Springer, p. 195-205.
[19] Han, J., Nichols, J.H., Rice, M., Klonoff, D.C.J.J.o.d.s., technology, 2020. The End of the Road for the YSI 2300 Analyzer: Where Do We Go Now? 14(3):595-600.
[20] Patergnani, S., Baldassari, F., De Marchi, E., Karkucinska-Wieckowska, A., Wieckowski, M.R., Pinton, P.J.M.i.e., 2014. Methods to monitor and compare mitochondrial and glycolytic ATP production. 542:313-332.
[21] Bhatt, D.P., Chen, X., Geiger, J.D., Rosenberger, T.A.J.J.o.C.B., 2012. A sensitive HPLC-based method to quantify adenine nucleotides in primary astrocyte cell cultures. 889:110-115.
[22] von Papen, M., Gambaryan, S., Schütz, C., Geiger, J.J.T.M., Hemotherapy, 2013. Determination of ATP and ADP secretion from human and mouse platelets by an HPLC assay. 40(2):109-116.
[23] Ni, Q., Mehta, S., Zhang, J.J.T.F.j., 2018. Live-cell imaging of cell signaling using genetically encoded fluorescent reporters. 285(2):203-219.
[24] O'Neil, R.G., Wu, L., Mullani, N.J.M.I., Biology, 2005. Uptake of a fluorescent deoxyglucose analog (2-NBDG) in tumor cells. 7(6):388-392.
[25] Tantama, M., Hung, Y.P., Yellen, G.J.J.o.t.A.C.S., 2011. Imaging intracellular pH in live cells with a genetically encoded red fluorescent protein sensor. 133(26):10034-10037.
[26] Burgstaller, S., Bischof, H., Gensch, T., Stryeck, S., Gottschalk, B., Ramadani-Muja, J., et al., 2019. pH-Lemon, a fluorescent protein-based pH reporter for acidic compartments. 4(4):883-891.
[27] Mohsin, M., Ahmad, A., Iqbal, M.J.B.l., 2015. FRET-based genetically-encoded sensors for quantitative monitoring of metabolites. 37(10):1919-1928.
[28] San Martín, A., Ceballo, S., Baeza-Lehnert, F., Lerchundi, R., Valdebenito, R., Contreras-Baeza, Y., et al., 2014. Imaging mitochondrial flux in single cells with a FRET sensor for pyruvate. 9(1):e85780.
[29] Bittner, C.X., Loaiza, A., Ruminot, I., Larenas, V., Sotelo-Hitschfe, T., Gutiérrez, R., et al., 2010. High resolution measurement of the glycolytic rate. 2:26.
[30] Berg, J., Hung, Y.P., Yellen, G.J.N.m., 2009. A genetically encoded fluorescent reporter of ATP: ADP ratio. 6(2):161-166.

[31] Matlashov, M.E., Bogdanova, Y.A., Ermakova, G.V., Mishina, N.M., Ermakova, Y.G., Nikitin, E.S., et al., 2015. Fluorescent ratiometric pH indicator SypHer2: Applications in neuroscience and regenerative biology. 1850(11):2318-2328.
[32] Iglesias, P., Penas, C., Barral-Cagiao, L., Pazos, E., Costoya, J.A.J.S.r., 2019. A bio-inspired hypoxia sensor using HIF1a-oxygen-dependent degradation domain. 9(1):1-7.
[33] Youssef, S., Ren, W., Ai, H.-w.J.A.c.b., 2016. A genetically encoded FRET sensor for hypoxia and prolyl hydroxylases. 11(9):2492-2498.
[34] Connolly, N.M., Theurey, P., Adam-Vizi, V., Bazan, N.G., Bernardi, P., Bolaños, J.P., et al., 2018. Guidelines on experimental methods to assess mitochondrial dysfunction in cellular models of neurodegenerative diseases. 25(3):542-572.
[35] Hung, Y.P., Yellen, G., 2014. Live-cell imaging of cytosolic NADH–NAD+ redox state using a genetically encoded fluorescent biosensor. Fluorescent protein-based biosensors. Springer, p. 83-95.
[36] Aguilera, K.Y., Brekken, R.A.J.B.-p., 2014. Hypoxia Studies with Pimonidazole in vivo. 4(19).
[37] Papkovsky, D.B., Zhdanov, A.V., 2015. Cell energy budget platform for assessment of cell metabolism. Mitochondrial Medicine: Volume II, Manipulating Mitochondrial Function:333.
[38] Takahashi, E., Yamaoka, Y.J.T.J.o.P.S., 2017. Simple and inexpensive technique for measuring oxygen consumption rate in adherent cultured cells. 67(6):731-737.
[39] Bénit, P., Chrétien, D., Porceddu, M., Yanicostas, C., Rak, M., Rustin, P.J.J.o.c.m., 2017. An effective, versatile, and inexpensive device for oxygen uptake measurement. 6(6):58.
[40] Dmitriev, R.I., Papkovsky, D.B.J.M., fluorescence, a.i., 2015. Intracellular probes for imaging oxygen concentration: how good are they? 3(3):034001.
[41] Wolfbeis, O.S.J.B., 2015. Luminescent sensing and imaging of oxygen: Fierce competition to the Clark electrode. 37(8):921-928.
[42] Romero, N., Rogers, G., Neilson, A., Dranka, B.J.A.T., Inc, 2018. Quantifying Cellular ATP Production Rate Using Agilent Seahorse XF Technology.
[43] Mookerjee, S.A., Gerencser, A.A., Nicholls, D.G., Brand, M.D.J.J.o.B.C., 2017. Quantifying intracellular rates of glycolytic and oxidative ATP production and consumption using extracellular flux measurements. 292(17):7189-7207.
[44] Yépez, V.A., Kremer, L.S., Iuso, A., Gusic, M., Kopajtich, R., Koňaříková, E., et al., 2018. OCR-Stats: Robust estimation and statistical testing of mitochondrial respiration activities using Seahorse XF Analyzer. 13(7):e0199938.
[45] Zhang, X., Yuan, T., Keijer, J., de Boer, V.C.J.b., 2021. OCRbayes: A Bayesian hierarchical modeling framework for Seahorse extracellular flux oxygen consumption rate data analysis.
[46] Iuso, A., Repp, B., Biagosch, C., Terrile, C., Prokisch, H., 2017. Assessing mitochondrial bioenergetics in isolated mitochondria from various mouse tissues using seahorse XF96 analyzer. Mitochondria. Springer, p. 217-230.
[47] Gnaiger, E.J.M.P.N.-. 2006. The Oxygraph for High-Resolution Respirometry.
[48] Li, Z., Graham, B.H., 2012. Measurement of mitochondrial oxygen consumption using a Clark electrode. Mitochondrial Disorders. Springer, p. 63-72.
[49] Silva, A.M., Oliveira, P.J., 2012. Evaluation of respiration with clark type electrode in isolated mitochondria and permeabilized animal cells. Mitochondrial Bioenergetics. Springer, p. 7-24.
[50] Ludikhuize, M.C., Meerlo, M., Burgering, B.M., Colman, M.J.R.J.S.p., 2021. Protocol to profile the bioenergetics of organoids using Seahorse. 2(1):100386.
[51] Russell, S., Wojtkowiak, J., Neilson, A., Gillies, R.J.J.S.r., 2017. Metabolic Profiling of healthy and cancerous tissues in 2D and 3D. 7(1):1-11.
[52] Kelbauskas, L., Glenn, H., Anderson, C., Messner, J., Lee, K.B., Song, G., et al., 2017. A platform for high-throughput bioenergy production phenotype characterization in single cells. 7(1):1-13.
[53] Kelbauskas, L., Ashili, S.P., Lee, K.B., Zhu, H., Tian, Y., Meldrum, D.R.J.S.r., 2018. Simultaneous multiparameter cellular energy metabolism profiling of small populations of cells. 8(1):1-12.
[54] Hai, P., Imai, T., Xu, S., Zhang, R., Aft, R.L., Zou, J., et al., 2019. High-throughput, label-free, single-cell photoacoustic microscopy of intratumoral metabolic heterogeneity. 3(5):381-391.
[55] Wang, Y., Zhao, N., Xiong, Y., Zhang, J., Zhao, D., Yin, Y., et al., 2020. Downregulated recycling process but not de novo synthesis of glutathione limits antioxidant capacity of erythrocytes in hypoxia. 2020.

[56] Hou, J., Wright, H.J., Chan, N.S.-K., Tran, R.D., Razorenova, O.V., Potma, E.O., et al., 2016. Correlating two-photon excited fluorescence imaging of breast cancer cellular redox state with seahorse flux analysis of normalized cellular oxygen consumption. 21(6):060503.
[57] Shah, A.T., Diggins, K.E., Walsh, A.J., Irish, J.M., Skala, M.C.J.N., 2015. In vivo autofluorescence imaging of tumor heterogeneity in response to treatment. 17(12):862-870.
[58] Walsh, A.J., Cook, R.S., Sanders, M.E., Aurisicchio, L., Ciliberto, G., Arteaga, C.L., et al., 2014. Quantitative optical imaging of primary tumor organoid metabolism predicts drug response in breast cancer. 74(18):5184-5194.
[59] Cannon, T.M., Shah, A.T., Walsh, A.J., Skala, M.C.J.J.o.b.o., 2015. High-throughput measurements of the optical redox ratio using a commercial microplate reader. 20(1):010503.
[60] Okkelman, I.A., Papkovsky, D.B., Dmitriev, R.I.J.C.P.A., 2020. Estimation of the mitochondrial membrane potential using fluorescence lifetime imaging microscopy. 97(5):471-482.
[61] Datta, R., Heaster, T.M., Sharick, J.T., Gillette, A.A., Skala, M.C.J.J.o.b.o., 2020. Fluorescence lifetime imaging microscopy: fundamentals and advances in instrumentation, analysis, and applications. 25(7):071203.
[62] Sen, R., Zhdanov, A.V., Bastiaanssen, T.F., Hirvonen, L.M., Svihra, P., Fitzgerald, P., et al., 2020. Mapping O 2 concentration in ex-vivo tissue samples on a fast PLIM macro-imager. 10(1):1-11.
[63] Witney, T.H., James, M.L., Shen, B., Chang, E., Pohling, C., Arksey, N., et al., 2015. PET imaging of tumor glycolysis downstream of hexokinase through noninvasive measurement of pyruvate kinase M2. 7(310):310ra169-310ra169.
[64] Qu, W., Oya, S., Lieberman, B.P., Ploessl, K., Wang, L., Wise, D.R., et al., 2012. Preparation and characterization of L- 5-11C -glutamine for metabolic imaging of tumors. Journal of nuclear medicine : official publication, Society of Nuclear Medicine 53(1):98.
[65] Chen, W., Silverman, D.H., Delaloye, S., Czernin, J., Kamdar, N., Pope, W., et al., 2006. 18F-FDOPA PET imaging of brain tumors: comparison study with 18F-FDG PET and evaluation of diagnostic accuracy. Journal of nuclear medicine : official publication, Society of Nuclear Medicine 47(6):904.
[66] Grassi, I., Nanni, C., Allegri, V., Morigi, J.J., Montini, G.C., Castellucci, P., et al., 2012. The clinical use of PET with 11C-acetate. 2(1):33.
[67] Clark, P.M., Flores, G., Evdokimov, N.M., McCracken, M.N., Chai, T., Nair-Gill, E., et al., 2014. Positron emission tomography probe demonstrates a striking concentration of ribose salvage in the liver. 111(28):E2866-E2874.
[68] Yao, R., Lecomte, R., Crawford, E.S.J.J.o.n.m.t., 2012. Small-animal PET: what is it, and why do we need it? 40(3):157-165.
[69] Driehuys, B., Nouls, J., Badea, A., Bucholz, E., Ghaghada, K., Petiet, A., et al., 2008. Small animal imaging with magnetic resonance microscopy. 49(1):35-53.
[70] Kobus, T., Wright, A.J., Scheenen, T.W., Heerschap, A.J.N.i.B., 2014. Mapping of prostate cancer by 1H MRSI. 27(1):39-52.
[71] Öz, G., Alger, J.R., Barker, P.B., Bartha, R., Bizzi, A., Boesch, C., et al., 2014. Clinical proton MR spectroscopy in central nervous system disorders. 270(3):658-679.
[72] Zhu, H., Edden, R.A.E., Ouwerkerk, R., Barker, P.B., 2011. High resolution spectroscopic imaging of GABA at 3 Tesla. Magnetic resonance in medicine 65(3):603.
[73] Hourani, R., Horská, A., Albayram, S., Brant, L.J., Melhem, E., Cohen, K.J., et al., 2006. Proton magnetic resonance spectroscopic imaging to differentiate between nonneoplastic lesions and brain tumors in children. Journal of Magnetic Resonance Imaging 23(2):99.
[74] Zhu, H., Soher, B.J., Ouwerkerk, R., Schär, M., Barker, P.B., 2013. Spin-echo magnetic resonance spectroscopic imaging at 7 T with frequency-modulated refocusing pulses. Magnetic resonance in medicine 69(5):1217.
[75] Liesenfeld, D.B., Habermann, N., Owen, R.W., Scalbert, A., Ulrich, C.M.J.C.e., biomarkers, p., 2013. Review of mass spectrometry–based metabolomics in cancer research. 22(12):2182-2201.
[76] Brown, M.V., McDunn, J.E., Gunst, P.R., Smith, E.M., Milburn, M.V., Troyer, D.A., et al., 2012. Cancer detection and biopsy classification using concurrent histopathological and metabolomic analysis of core biopsies. Genome medicine 4(4):33.
[77] Denkert, C., Budczies, J., Kind, T., Weichert, W., Tablack, P., Sehouli, J., et al., 2006. Mass spectrometry-based metabolic profiling reveals different metabolite patterns in invasive ovarian carcinomas and ovarian borderline tumors. Cancer research 66(22):10795.

[78] Hori, S., Nishiumi, S., Kobayashi, K., Shinohara, M., Hatakeyama, Y., Kotani, Y., et al., 2011. A metabolomic approach to lung cancer. Lung cancer (Amsterdam, Netherlands) 74(2):284.

[79] Song, H., Wang, L., Liu, H.-L., Wu, X.-B., Wang, H.-S., Liu, Z.-H., et al., 2011. Tissue metabolomic fingerprinting reveals metabolic disorders associated with human gastric cancer morbidity. Oncology reports 26(2):431.

[80] Denkert, C., Budczies, J., Weichert, W., Wohlgemuth, G., Scholz, M., Kind, T., et al., 2008. Metabolite profiling of human colon carcinoma–deregulation of TCA cycle and amino acid turnover. Molecular cancer 7(1):72.

[81] Yuneva, M.O., Fan, T.W.M., Allen, T.D., Higashi, R.M., Ferraris, D.V., Tsukamoto, T., et al., 2012. The metabolic profile of tumors depends on both the responsible genetic lesion and tissue type. Cell metabolism 15(2):157.

[82] Hensley, C.T., Faubert, B., Yuan, Q., Lev-Cohain, N., Jin, E., Kim, J., et al., 2016. Metabolic heterogeneity in human lung tumors. Cell 164(4):681.

[83] Cogliati, S., Enriquez, J.A., Scorrano, L.J.T.i.b.s., 2016. Mitochondrial cristae: where beauty meets functionality. 41(3):261-273.

[84] Jiang, Y.-f., Lin, S.-s., Chen, J.-m., Tsai, H.-z., Hsieh, T.-s., Fu, C.-y.J.S.r., 2017. Electron tomographic analysis reveals ultrastructural features of mitochondrial cristae architecture which reflect energetic state and aging. 7(1):1-11.

[85] Moscheni, C., Malucelli, E., Castiglioni, S., Procopio, A., De Palma, C., Sorrentino, A., et al., 2019. 3D quantitative and ultrastructural analysis of mitochondria in a model of doxorubicin sensitive and resistant human colon carcinoma cells. 11(9):1254.

[86] Tobias, I., Khazaee, R., Betts, D.J.C.p.i.s.c.b., 2018. Analysis of mitochondrial dimensions and cristae structure in pluripotent stem cells using transmission electron microscopy. 47(1):e67.
