# Supplementary material for: Metabolic therapy and bioenergetic analysis: The missing piece of the puzzle
Source: Mol Metab. 2021 Nov 5;54:101389. doi: 10.1016/j.molmet.2021.101389 (PMC8637646; doi:10.1016/j.molmet.2021.101389)
Supplement: Multimedia component 3 [file mmc3.pdf]

Cell line (XF Real-Time ATP Assay)	glycoATP (≈ %)		mitoATP (≈ %)		
	Mean	SD	Mean	SD	Reference
(brain) Human Astrocytes	75.0		25.0		[1]
(brain) Glioblastoma stem cells (GSCs; 8.11)	35.0		65.0		[1]
(brain) Glioblastoma stem cells (GSCs; 272)	23.0		77.0		[1]
(brain) Glioblastoma stem cells (GSCs; 6.27)	17.0		83.0		[1]
(pancreas) PANC-1	15.7	2.1	83.1	3.1	[2]
(breast) BT-474	6.9	2.6	91.5	3.9	[2]
(cervix) HeLa	20.9	3.3	77.7	0.1	[2]
(breast) ZR-75	27.7	3.4	72.0	4.8	[2]
(colon) HCT 116	28.9	6.0	71.0	5.0	[2]
(skin) HMEC	58.9	10.4	40.5	2.5	[2]
(breast, metastatic) SKBR3	71.2	4.8	28.2	3.8	[2]
(breast) BT549	60.1	9.7	40.0	5.7	[2]
(colon) HT-29	55.4	14.6	43.3	8.3	[2]
(breast) T-47D	49.0	6.5	50.6	3.3	[2]
(ovary) SK-OV-3	19.1	4.6	80.9	6.8	[2]
(breast) Hs 578T	79.4	13.8	21.5	2.5	[2]
(breast) MCF 10A	63.8	17.6	35.1	5.2	[2]
(peripheral blood; promyeloblast) HL-60	40.0	14.5	60.3	0.0	[2]
(peripheral blood, T lymphocyte) Jukart	63.7	0.0	37.3	4.6	[2]
(skin) A-431	59.0	8.9	41.0	3.1	[2]
(breast) MCF7	31.3	10.4	68.7	7.5	[2]
(peripheral blood) RAW264.7	28.0		72.0		[3]
(fibroblast) Primary mouse embryonic fibroblasts (MEFs)	20.5	6.8	79.5	14.6	[4]
(kidney) HEK293	34.0		66.0		[5]
(lung) A549	43.0	8.1	57.8	15.0	[6]
(lung) H460	44.9	4.3	54.8	4.4	[6]
(lung) NCI-H1975	28.4	0.0	70.5	7.2	[6]
(lung) PC-9	28.1		71.5		[6]
(gastric) SGC-7901	42.0	3.6	57.8	5.0	[7]
(gastric) MGC-803	43.5	4.0	56.9	3.0	[7]
(breast) MDA-MB-468	33.9		66.1		[8]
(breast) MDA-MB-231	63.4		36.6		[8]
(breast) HME1	71.3		28.7		[8]
(breast) HS578T	66.0		34.0		[8]
(breast) HCC70	50.4		49.6		[8]
(brain) iPSC-derived motor neurons (2dpi)	64.9	3.0	34.9	3.0	[9]
(brain) iPSC-derived motor neurons (4dpi)	10.0	2.9	90.3	10.6	[9]
(other) Adipose MSC	81.5	7.7	18.9	1.3	[10]
(other) Umbilical MSC	74.6	4.1	27.1	2.7	[10]

**(kidney) UOK262**	97.0	4.3	3.0	5.7	[11]
**(breast) MCF7**	20.9	3.5	81.1	30.8	[12]
**(breast) MCF7-IGF2**	41.0	7.0	59.3	9.5	[12]
**(peripheral blood) MDSCs**	66.3		32.3		[13]
**(cervix) HeLa**	63.9	7.0	37.7	3.3	[14]
**(peripheral blood) Primary human macrophages**	19.0		81.0		[15]
**(liver) HepG2**	36.8	3.8	63.2	6.4	[16]
**(other) human adipose stem cells (hASCs)-1**	27.3	2.5	72.9	15.0	[17]
**(other) human adipose stem cells (hASCs)-2**	40.6	9.2	59.4	24.7	[17]
**(other) human adipose stem cells (hASCs)-3**	71.8	0.8	29.2	2.9	[17]
**(other) human definitive endodermal progenitor cells (hEPSC)-1**	36.6	7.6	63.4	11.0	[17]
**(peripheral blood) THP-1**	26.7	3.5	72.1	4.4	[18]
**(peripheral blood) hMDM - Human M1 Macrophages**	26.1	5.2	73.9	9.0	[18]
**(kidney) 786-O**	81.2		18.8		[19]
**(brain) U87MG**	56.4		43.6		[20]
**(brain) U87MG**	89.6		10.3		[21]
**(brain) U251**	76.9		23.0		[21]
**(brain) LN18**	77.4		22.5		[21]
**(brain) SF767**	74.7		25.2		[21]
**(brain) Glioblastoma stem cells; GBM18**	65.2		34.8		[20]
**(brain) Glioblastoma stem cells; GBM27**	48.3		51.7		[20]
**(brain) Glioblastoma stem cells; GBM38**	74.8		25.2		[20]
**(bladder) T24**	47.6	22.0	52.4	17.7	[22]
**(bladder) 5637**	54.3	18.2	45.7	16.4	[22]
**(bladder) HT-1376**	55.0	19.5	45.0	14.5	[22]
**(bladder) HB-CLS-2**	64.6	10.9	35.4	5.5	[22]
**(bladder) TCC-SUP**	60.2	8.7	39.8	4.4	[22]
**(bladder) CLS-439**	89.5	11.3	10.5	2.4	[22]
**(fibroblast) 3T3-L1 (non-differentiation medium)**	16.4	2.7	83.1	7.1	[23]
**(fibroblast) 3T3-L1 (adipogenic medium)**	19.5	2.2	83.2	4.4	[23]
**(breast) BT-474 cells**	13.9	2.7	84.3	8.8	[24]
**(breast) BT-474 cells**	42.8	5.2	57.2	28.9	[24]
**(lung) A549**	41.8		58.2		[25]
**(prostate) DU145**	76.3		23.7		[25]
**(colon) DiFi cells**	34.8	1.6	64.5	2.3	[26]
**(liver) HEPG2**	68.8	13.0	31.5	28.5	[27]
**(liver) HuH-7**	67.6	11.6	32.4	20.4	[27]
**(liver) HEPG2**	46.0	8.0	54.4	7.3	[28]

**(peripheral blood) Jurkat**	60.1		39.9		[29]
**(endothelial) HUVEC (no glucose)**	82.1	17.6	18.5	4.9	[30]
**(endothelial) HUVEC (5mM glucose)**	96.7	21.6	3.6	1.9	[30]
**(fibroblast) primary dermal fibroblasts**	33.9		65.5		[31]
**(liver) HuH-7**	44.8		55.6		[32]
**(pancreas) MIA PaCa-2**	73.2	9.5	27.9	6.2	[33]
**(pancreas) MIA PaCa-2**	74.7	6.2	26.9	5.6	[33]
**(colon) DLD-1**	58.6	4.4	41.5	5.3	[34]
**(muscle) Primary skeletal muscle (mice)**	37.7		62.3		[35]
**(skin) HaCaT**	35.6		63.6		[36]
**(pharynx) FaDu**	36.3	4.2	63.7	5.2	[36]
**(pharynx; metastatic) Detroit562**	28.7	4.0	73.4	6.1	[36]
**(fibroblast) HGFb cell line**	16.1		83.5		[36]
**(fibroblast) Patient-derived healthy fibroblasts (PD-fibroblasts)**	33.7		67.0		[36]
**(fibroblast) Patient derived-CAFs (PD-CAFs)**	26.4		73.1		[36]
**(other) human adipose tissue-derived stem cells (hASCs); HC016-1**	97.7	5.2	2.3	0.2	[37]
**(other) human adipose tissue-derived stem cells (hASCs); HC016-2**	97.4	1.7	2.6	0.1	[37]
**(brain) WT-immortalized astrocytes (WT-iAstro)**	33.7	10.7	66.3	11.4	[38]
**(fibroblast) L929**	12.8	10.2	87.0	4.4	[39]
**(fibroblast) L929 p0**	2.3		97.7	7.6	[39]
**(breast) MCF10a**	27.9		72.8		[40]
**(breast) T-47D**	10.6		89.7		[40]
**(breast) MCF7**	9.7		90.5		[40]
**(breast) MDA-MB-175-VII**	3.5		96.4		[40]
**(breast) BT-474**	16.7		83.0		[40]
**(breast) HCC1419**	5.7		94.4		[40]
**(breast) SKBR3**	2.6		96.9		[40]
**(breast) HBL-100**	25.0		75.8		[40]
**(breast) Hs578T**	55.4		45.2		[40]
**(breast) BT549**	21.1		78.8		[40]
**(breast) MDA-MB-231(NI)**	20.1		79.8		[40]
**(breast) ESH-172**	42.3		58.3		[40]
**(pancreas) MIA PaCa-2**	13.1	1.5	87.4	3.6	[41]
**(breast) MCF7**	25.9	2.3	75.5	5.2	[42]

Table S2. Summary data from Seahorse XF Real-Time ATP Rate Assay (glycoATP/mitoATP). We have explored the Agilent Cell Analysis Publication Database [43; 44] for publications using the Real-Time ATP Rate Assay in the period of 2015-2021. Furthermore, Google Scholar databases were manually searched with the following syntax to improve results: “mitoATP” “Seahorse”; “glycoATP” “Seahorse”; “ATP Rate” “Seahorse” (last 5 years). Publications were screened for written data in text

and, where not available, percentages were estimated semi-automatically using ImageJ [45]. Briefly, high-resolution PDF figures were transformed into ".tiff" image format and pre-processed in ImageJ using the "Find Edges" and "Find Maxima" plugins. After detecting suitable points or manually adjusting them, the scale factors provided by the authors were used as reference by the "Set Scale" function, and measurements of the length of lines were measured using the "Measure" function. Results were in less than ≈ 2% of error when comparing independent measurements and with the data provided in some publications. In this section, we want to underscore that these values illustrate a relative comparison, not concrete flux rates from individual experiments. To compare absolute values in different experimental conditions, they would have to be arbitrarily relativized to a specific parameter, such as basal respiration, and subsequently expressed as a percentage of change after injection of mitochondrial inhibitors. This would constitute a very crude way of describing mitochondrial function, while ignoring, unavoidably, the basal metabolic activity of each cell type (flux rate per normalization unit). As an example, OCR-changes upon stress in maximal and spare respiratory capacity of normal astrocytes is significantly higher (up to 150% on average) than that of established U87MG glioblastoma cells [46-57]. This reveals partial mitochondrial dysfunction or resistance to mitochondrial inhibitors in U87MG cells but cannot directly define the basal metabolic activity of these cell lines. Empty cells indicate data was not given in the original work or could not be estimated.

References:

[1] Puca, F., Yu, F., Bertolacci, C., Pettazzoni, P., Carugo, A., Huang-Hobbs, E., et al., 2020. Medium-chain acyl-CoA dehydrogenase, a gatekeeper of mitochondrial function in glioblastoma multiforme.

[2] Romero, N., Swain, P.M., Kam, Y., Rogers, G., Dranka, B.P., 2018. Bioenergetic profiling of cancer cell lines: quantifying the impact of glycolysis on cell proliferation, Cancer research. AMER ASSOC CANCER RESEARCH 615 CHESTNUT ST, 17TH FLOOR, PHILADELPHIA, PA ….

[3] Passalacqua, K.D., Lu, J., Goodfellow, I., Kolawole, A.O., Arche, J.R., Maddox, R.J., et al., 2019. Glycolysis is an intrinsic factor for optimal replication of a norovirus. 10(2).

[4] Pacelli, C., Adipietro, I., Malerba, N., Squeo, G.M., Piccoli, C., Amoresano, A., et al., 2020. Loss of Function of the Gene Encoding the Histone Methyltransferase KMT2D Leads to Deregulation of Mitochondrial Respiration. 9(7):1685.

[5] Cilenti, L., Di Gregorio, J., Ambivero, C.T., Andl, T., Liao, R., Zervos, A.S.J.S.r., 2020. Mitochondrial MUL1 E3 ubiquitin ligase regulates Hypoxia Inducible Factor (HIF-1α) and metabolic reprogramming by modulating the UBXN7 cofactor protein. 10(1):1-15.

[6] Swain, P., Romero, N., Kam, Y., Dranka, B.P., 2019. Differential use of lactate for mitochondria respiration by NSCLC cells. AACR.

[7] Wang, H., Luo, J., Tian, W., Yan, W., Ge, S., Zhang, Y., et al., 2019. γ-Tocotrienol inhibits oxidative phosphorylation and triggers apoptosis by inhibiting mitochondrial complex I subunit NDUFB8 and complex II subunit SDHB. 417:42-53.

[8] Lanning, N.J., Castle, J.P., Singh, S.J., Leon, A.N., Tovar, E.A., Sanghera, A., et al., 2017. Metabolic profiling of triple-negative breast cancer cells reveals metabolic vulnerabilities. 5(1):1-14.

[9] Pourshafie, N., Masati, E., Bunker, E., Nickolls, A.R., Thepmankorn, P., Johnson, K., et al., 2020. Linking epigenetic dysregulation, mitochondrial impairment, and metabolic dysfunction in SBMA motor neurons. 5(13).

[10] Marcoccia, R., Nesci, S., Merlo, B., Ballotta, G., Algieri, C., Pagliarani, A., et al., 2021. Biological characteristics and metabolic profile of canine mesenchymal stem cells isolated from adipose tissue and umbilical cord matrix. 16(3):e0247567.

[11] Madala, H.R., Helenius, I.T., Zhou, W., Mills, E., Zhang, Y., Liu, Y., et al., 2020. Nitrogen trapping as a therapeutic strategy in tumors with mitochondrial dysfunction. 80(17):3492-3506.

[12] Vella, V., Nicolosi, M.L., Giuliano, M., Morrione, A., Malaguarnera, R., Belfiore, A.J.C., 2019. Insulin receptor isoform A modulates metabolic reprogramming of breast cancer cells in response to IGF2 and insulin stimulation. 8(9):1017.

[13] Neamah, W.H., Singh, N.P., Alghetaa, H., Abdulla, O.A., Chatterjee, S., Busbee, P.B., et al., 2019. AhR activation leads to massive mobilization of myeloid-derived suppressor cells with

immunosuppressive activity through regulation of CXCR2 and MicroRNA miR-150-5p and miR-543-3p that target anti-inflammatory genes. 203(7):1830-1844.
[14] Salewskij, K., Rieger, B., Hager, F., Arroum, T., Duwe, P., Villalta, J., et al., 2020. The spatio-temporal organization of mitochondrial F1FO ATP synthase in cristae depends on its activity mode. 1861(1):148091.
[15] Mahon, O.R., Kelly, D.J., McCarthy, G., Dunne, A.J.O., cartilage, 2020. Osteoarthritis-associated basic calcium phosphate crystals alter immune cell metabolism and promote M1 macrophage polarization. 28(5):603-612.
[16] Park, S., Kim, K., Haam, G.-O., Ban, H.S., Kim, J.-A., Park, B.C., et al., 2021. Long-term depletion of Cereblon induces mitochondrial dysfunction in cancer cells.
[17] Ma, Y., Ma, M., Sun, J., Li, W., Li, Y., Guo, X., et al., 2019. CHIR-99021 regulates mitochondrial remodelling via β-catenin signalling and miRNA expression during endodermal differentiation. 132(15).
[18] Cumming, B.M., Addicott, K.W., Adamson, J.H., Steyn, A.J.J.E., 2018. Mycobacterium tuberculosis induces decelerated bioenergetic metabolism in human macrophages. 7:e39169.
[19] Dey, P., Son, J.Y., Kundu, A., Kim, K.S., Lee, Y., Yoon, K., et al., 2019. Knockdown of pyruvate kinase M2 inhibits cell proliferation, metabolism, and migration in Renal CELL carcinoma. 20(22):5622.
[20] Duraj, T., García-Romero, N., Carrión-Navarro, J., Madurga, R., Mendivil, A.O.d., Prat-Acin, R., et al., 2021. Beyond the Warburg Effect: Oxidative and Glycolytic Phenotypes Coexist within the Metabolic Heterogeneity of Glioblastoma. 10(2):202.
[21] Sesen, J., Dahan, P., Scotland, S.J., Saland, E., Dang, V.-T., Lemarié, A., et al., 2015. Metformin inhibits growth of human glioblastoma cells and enhances therapeutic response. 10(4):e0123721.
[22] Oresta, B., Pozzi, C., Braga, D., Hurle, R., Lazzeri, M., Colombo, P., et al., 2021. Mitochondrial metabolic reprogramming controls the induction of immunogenic cell death and efficacy of chemotherapy in bladder cancer. 13(575).
[23] Fujiwara, M., Tian, L., Le, P.T., DeMambro, V.E., Becker, K.A., Rosen, C.J., et al., 2019. The mitophagy receptor Bcl-2–like protein 13 stimulates adipogenesis by regulating mitochondrial oxidative phosphorylation and apoptosis in mice. 294(34):12683-12694.
[24] Papadopoli, D.J., Ma, E.H., Roy, D., Russo, M., Bridon, G., Avizonis, D., et al., 2020. Methotrexate elicits pro-respiratory and anti-growth effects by promoting AMPK signaling. 10(1):1-9.
[25] Xie, J., Ye, J., Cai, Z., Luo, Y., Zhu, X., Deng, Y., et al., 2020. GPD1 Enhances the Anticancer Effects of Metformin by Synergistically Increasing Total Cellular Glycerol-3-Phosphate. 80(11):2150-2162.
[26] Lorenzato, A., Magrì, A., Matafora, V., Audrito, V., Arcella, P., Lazzari, L., et al., 2020. Vitamin C restricts the emergence of acquired resistance to EGFR-targeted therapies in colorectal cancer. 12(3):685.
[27] Tripodi, F., Badone, B., Vescovi, M., Milanesi, R., Nonnis, S., Maffioli, E., et al., 2020. Methionine Supplementation Affects Metabolism and Reduces Tumor Aggressiveness in Liver Cancer Cells. 9(11):2491.
[28] Espinosa, J.A., Pohan, G., Arkin, M.R., Markossian, S.J.C.P., 2021. Real-Time Assessment of Mitochondrial Toxicity in HepG2 Cells Using the Seahorse Extracellular Flux Analyzer. 1(3):e75.
[29] Kory, N., uit de Bos, J., van der Rijt, S., Jankovic, N., Güra, M., Arp, N., et al., 2020. MCART1/SLC25A51 is required for mitochondrial NAD transport. 6(43):eabe5310.
[30] Moessinger, C., Nilsson, I., Muhl, L., Zeitelhofer, M., Heller Sahlgren, B., Skogsberg, J., et al., 2020. VEGF-B signaling impairs endothelial glucose transcytosis by decreasing membrane cholesterol content. 21(7):e49343.
[31] Gropman, A., Uittenbogaard, M., Brantner, C.A., Wang, Y., Wong, L.-J., Chiaramello, A.J.M.G., et al., 2020. Molecular genetic and mitochondrial metabolic analyses confirm the suspected mitochondrial etiology in a pediatric patient with an atypical form of alternating hemiplegia of childhood. 24:100609.
[32] Sasaki, K., Nishina, S., Yamauchi, A., Fukuda, K., Hara, Y., Yamamura, M., et al., 2021. Nanoparticle-Mediated Delivery of 2-Deoxy-D-Glucose Induces Antitumor Immunity and Cytotoxicity in Liver Tumors in Mice. 11(3):739-762.

[33] Sritangos, P., Pena Alarcon, E., James, A.D., Sultan, A., Richardson, D.A., Bruce, J.I.J.C., 2020. Plasma membrane Ca2+ atpase isoform 4 (PMCA4) has an important role in numerous hallmarks of pancreatic cancer. 12(1):218.
[34] Zhang, W., Sviripa, V.M., Kril, L.M., Yu, T., Xie, Y., Hubbard, W.B., et al., 2019. An underlying mechanism of dual Wnt inhibition and AMPK activation: mitochondrial uncouplers masquerading as Wnt inhibitors. 62(24):11348-11358.
[35] Leber, A., Hontecillas, R., Tubau-Juni, N., Zoccoli-Rodriguez, V., Goodpaster, B., Bassaganya-Riera, J.J.S.r., 2020. Abscisic acid enriched fig extract promotes insulin sensitivity by decreasing systemic inflammation and activating LANCL2 in skeletal muscle. 10(1):1-9.
[36] Zima, T., Králové, H., 2020. New Frontiers in the Research of Ph. D. Students.
[37] Garrido-Pascual, P., Alonso-Varona, A., Castro, B., Burón, M., Palomares, T.J.S.c.r., therapy, 2020. H 2 O 2-preconditioned human adipose-derived stem cells (HC016) increase their resistance to oxidative stress by overexpressing Nrf2 and bioenergetic adaptation. 11(1):1-14.
[38] Dematteis, G., Vydmantaitė, G., Ruffinatti, F.A., Chahin, M., Farruggio, S., Barberis, E., et al., 2020. Proteomic analysis links alterations of bioenergetics, mitochondria-ER interactions and proteostasis in hippocampal astrocytes from 3xTg-AD mice. 11(8):1-16.
[39] Dawson, E.R., Patananan, A.N., Sercel, A.J., Teitell, M.A.J.S.r., 2020. Stable retention of chloramphenicol-resistant mtDNA to rescue metabolically impaired cells. 10(1):1-14.
[40] Martin, S.D., McGee, S.L.J.C., metabolism, 2019. A systematic flux analysis approach to identify metabolic vulnerabilities in human breast cancer cell lines. 7(1):1-14.
[41] Huang, C., Lan, W., Fraunhoffer, N., Meilerman, A., Iovanna, J., Santofimia-Castaño, P.J.C., 2019. Dissecting the anticancer mechanism of trifluoperazine on pancreatic ductal adenocarcinoma. 11(12):1869.
[42] Kam, Y., Romero, N., Swain, P., Dranka, B.P., 2017. Characterization of fuel dependencies in multidrug resistant breast cancer cells, Presented at the American Association of Cancer Researchers Annual Meeting.
[43] Cell, A.S.X.H.T., Kit, A.A., 2020. Agilent Seahorse XF Hu T Cell Activation Assay Kit.
[44] Leung, D.T., Chu, S., 2018. Measurement of oxidative stress: mitochondrial function using the seahorse system. Preeclampsia. Springer, p. 285-293.
[45] Abràmoff, M.D., Magalhães, P.J., Ram, S.J.J.B.i., 2004. Image processing with ImageJ. 11(7):36-42.
[46] Staricha, K., Meyers, N., Garvin, J., Liu, Q., Rarick, K., Harder, D., et al., 2020. Effect of high glucose condition on glucose metabolism in primary astrocytes. 1732:146702.
[47] Li, W., Choudhury, G.R., Winters, A., Prah, J., Lin, W., Liu, R., et al., 2018. Hyperglycemia alters astrocyte metabolism and inhibits astrocyte proliferation. 9(4):674.
[48] Thevenet, J., De Marchi, U., Domingo, J.S., Christinat, N., Bultot, L., Lefebvre, G., et al., 2016. Medium-chain fatty acids inhibit mitochondrial metabolism in astrocytes promoting astrocyte-neuron lactate and ketone body shuttle systems. 30(5):1913-1926.
[49] Potter, P.G.W., Walker, J.M.V., Robb, J.L., Chilton, J.K., Williamson, R., Randall, A., et al., 2018. Human primary astrocytes increase basal fatty acid oxidation following recurrent low glucose to maintain intracellular nucleotide levels.271981.
[50] Prabhu, A., Sarcar, B., Miller, C.R., Kim, S.-H., Nakano, I., Forsyth, P., et al., 2015. Ras-mediated modulation of pyruvate dehydrogenase activity regulates mitochondrial reserve capacity and contributes to glioblastoma tumorigenesis. 17(9):1220-1230.
[51] Li, J., Liu, Q., Liu, Z., Xia, Q., Zhang, Z., Zhang, R., et al., 2018. KPNA2 promotes metabolic reprogramming in glioblastomas by regulation of c-myc. 37(1):1-15.
[52] Massalha, W., Markovits, M., Pichinuk, E., Feinstein-Rotkopf, Y., Tarshish, M., Mishra, K., et al., 2019. Minerval (2-hydroxyoleic acid) causes cancer cell selective toxicity by uncoupling oxidative phosphorylation and compromising bioenergetic compensation capacity. 39(1).
[53] Rinaldi, L., Sepe, M., Delle Donne, R., Conte, K., Arcella, A., Borzacchiello, D., et al., 2017. Mitochondrial AKAP1 supports mTOR pathway and tumor growth. 8(6):e2842-e2842.
[54] Chen, Z., Li, S., Shen, L., Wei, X., Zhu, H., Wang, X., et al., 2020. NF-kappa B interacting long noncoding RNA enhances the Warburg effect and angiogenesis and is associated with decreased survival of patients with gliomas. 11(5):1-18.

[55] Desai, V., Jain, A., Shaghaghi, H., Summer, R., Lai, J.C., Bhushan, A.J.A.r., 2019. Combination of biochanin A and temozolomide impairs tumor growth by modulating cell metabolism in glioblastoma multiforme. 39(1):57-66.
[56] Kim, J., Han, J., Jang, Y., Kim, S.J., Lee, M.J., Ryu, M.J., et al., 2015. High-capacity glycolytic and mitochondrial oxidative metabolisms mediate the growth ability of glioblastoma. 47(3):1009-1016.
[57] Grigalavicius, M., Mastrangelopoulou, M., Arous, D., Juzeniene, A., Ménard, M., Skarpen, E., et al., 2020. Photodynamic efficacy of cercosporin in 3D tumor cell cultures. 96(3):699-707.
