# Supplementary material for: Metabolic therapy and bioenergetic analysis: The missing piece of the puzzle
Source: Mol Metab. 2021 Nov 5;54:101389. doi: 10.1016/j.molmet.2021.101389 (PMC8637646; doi:10.1016/j.molmet.2021.101389)
Supplement: Multimedia component 4 [file mmc4.pdf]

	Seahorse “Mito Fuel Flex” or “Substrate Oxidation Stress Test” Assays	Pyruvate Dependency (≈ %)		Glutamine Dependency (≈ %)		Fatty acids Dependency (≈ %)		Pyruvate Capacity (≈ %)		Glutamine Capacity (≈ %)		Fatty acids Capacity (≈ %)		Pyruvate Flexibility (≈ %)		Glutamine Flexibility (≈ %)		Fatty acids Flexibility (≈ %)		Glutamine/ Fatty Acids Dependency (≈ %)		Glutamine/ Fatty Acids Capacity (≈ %)		Glutamine/Fatty Acids Dependency Flexibility (≈ %)	
**References**	**Cell line**	**Mean**	**SD**	**Mean**	**SD**	**Mean**	**SD**	**Mean**	**SD**	**Mean**	**SD**	**Mean**	**SD**	**Mean**	**SD**	**Mean**	**SD**	**Mean**	**SD**	**Mean**	**SD**	**Mean**	**SD**	**Mean**	**SD**
[1]	(bladder) RT112	60.5	3.5	0.0	0.0	3.8	14.2	99.8		16.6		18.5		39.3	1.0	16.6	2.2	14.7	14.8						
[1]	(bladder) 5637	33.8	2.1	0.0	0.0	12.1	6.8	83.3		55.3		54.9		49.5	4.8	55.3	1.6	42.8	7.6						
[2]	(breast) BRCA1 (185delAG/+) MCF10A (control)	34.7	2.2	36.1	2.5			42.5	3.3	29.7	3.3			8.0	4.1	6.9	3.9								
[2]	(breast) BRCA1 (185delAG/+) MCF10A (metformin)	37.1	2.2	39.7	1.8			34.7	3.9	20.8	1.5			-2.9	4.5	19.8	2.6								
[3]	(adipocyte) T37i brown adipocytes (control)	29.1	7.3	11.3	5.2	71.6	3.8	9.9	5.7	2.4	2.7	70.8	4.5	-19.2		-8.9		-0.8							
[3]	(adipocyte) T37i brown adipocytes (CL induced)	28.9	8.5	9.6	6.6	66.0	5.6	33.5	7.5	16.2	2.2	63.9	9.4	4.6		6.5		-2.0							
[4]	(muscle) C2C12 control	40.8	2.8	18.1	6.0	40.8	3.5																		
[5]	(muscle) C2C12			11.2	1.4	21.2	2.3																		
[6]	(fibroblast) Cardiac fibroblast	27.8	12.0	1.5	1.0	108.4	25.4																		
[6]	(esophagus) oesophageal cancer cells (KYSE)	57.4	5.7	55.3	3.4	1.0	1.0																		
[7]	(colon) CT26.WT	37.6	3.6	11.6	5.9	29.0	6.3	76.2		21.5		38.6		38.6	5.0	9.9	8.6	9.6	6.3						
[8]	(fibroblast) Late passage human fibroblast	40.0	9.6					44.9	7.9					4.9						53.6	7.3	59.1	8.9	5.6	
[8]	(fibroblast) Early passage human fibroblast	16.2	6.8					68.1	4.8					52.0						30.8	5.3	78.7	6.3	47.9	
[8]	(fibroblast) Late passage human fibroblast	54.0	9.9					55.2	7.3					1.2						46.1	8.0	97.4	12.2	51.3	
[8]	(fibroblast) Late passage human fibroblast + Rapamycin	49.0	9.1					94.8	5.8					45.8						17.5	5.2	90.9	11.5	73.4	
[9]	(muscle) Primary myotubes	54.3	8.8			56.8	8.8	60.9	9.9			43.4	7.3	6.6				-13.4							
[9]	(muscle) Primary myotubes	30.1	2.4			21.3	2.9	62.7	3.3			58.7	4.4	32.6				37.5							
[10]	(gastric) GIST	28.9	3.9	0.0	0.0	40.8	2.0	37.0		19.0		59.9		8.1	15.6	19.0	2.7	19.1	3.6						
[11]	(peripheral blood) MDSCs 1%	71.4	9.5	-10.3	33.7	137.3	45.0																		
[11]	(peripheral blood) MDSCs 21%	12.5	13.4	45.3	7.8	29.6	21.7																		
[11]	(colon) HCT116 1%02	71.5	3.8	32.5	3.9	26.1	2.7																		
[11]	(colon) HCT116 1%02	68.1	4.8	31.6	1.5	23.7	3.3																		
[11]	(colon) HCT116 21%02	39.2	6.0	22.9	22.9	55.9	2.6																		
[11]	(colon) HCT116 21%02	44.5	2.9	24.9	2.6	70.4	3.6																		
[12]	(colon) HCT116	35.2	2.5	16.7	2.6	41.1	1.8	81.2		88.7		88.8		46.0	3.7	72.0	8.1	47.7	3.3						
[13]	(cervix) HeLa (glucose media)	35.6	2.9			27.4	3.4	101.2				75.7		65.6	3.6			48.2	4.6						

[13]	(cervix) HeLa (galactose media)	19.4	6.3			0.0	0.0	102.6				82.3		83.2	6.2			82.3	4.7						
[14]	(kidney) HEK293	14.8	3.5	26.6	1.7	76.7	2.7																		
[15]	(cervix) HeLa			33.1	2.7																				
[15]	(liver) HepG2			18.1	18.1																				
[16]	(cervix) HeLa	32.9	1.4	46.8	3.8	7.4	4.4	26.2	2.0	42.1	2.6	43.5	4.1	-6.7		-4.8		36.1							
[17]	(liver) AML12 hepatocytes	29.1	1.8	18.3	1.4	53.7	1.9	31.9	2.5	11.5	1.5	57.9	1.6	2.8		-6.8		4.2							
[18]	(liver) Hepg2	59.1	10.1	26.8	5.0	54.1	11.1																		
[19]	(breast) Human mammary epithelial (HME) cells			36.1	6.0																				
[19]	(breast) HME-LT			58.1	17.1																				
[19]	(breast) HME-PR			49.0	4.1																				
[20]	(endothelial) HUVEC (non-tip cells)	18.1	3.2	32.1	2.0	69.1	1.2	13.5	2.4	14.9	4.2	66.7	3.4	-4.6		-17.2		-2.4							
[20]	(endothelial) HUVEC (tip cells)	26.5	3.8	42.0	2.4	63.3	1.2	23.9	3.2	33.6	7.7	59.4	2.8	-2.6		-8.5		-4.0							
[21]	(brain) LN229	44.9	6.7	20.3	10.9	69.5	15.2																		
[22]	(endothelial) Primary lung endothelial vascular cells (LMVEC) (air)	60.0	4.9	12.6	2.3	19.2	3.1																		
[22]	(endothelial) Primary lung endothelial vascular cells (LMVEC) (hyperoxia)	59.4	3.0	16.4	16.4	11.4	2.2																		
[23]	(fibroblast) Mouse Embryonic Fibroblasts (MEFs)	31.0	2.3	1.6	2.5			88.5		55.8				57.6	3.0	54.3	3.3								
[24]	(skin) Human melanoma	43.6	4.4	21.1	1.4	5.5	4.1																		
[24]	(skin) Human melanoma	43.5	4.3	21.6	1.7	5.2	4.4	71.4		33.7		41.6		27.9	9.8	12.1	2.6	36.4	4.5						
[25]	(brain) Primary microglia	31.1	2.0	39.9	5.4	33.8	6.6																		
[26]	(brain) SK-N-AS human neuroblastoma	44.7	2.1	3.6	2.0	34.4	2.4	72.5	3.4	33.0	1.9	54.0	2.2	26.7		30.2		19.4							
[27]	(peripheral blood) Macrophages (normal fat diet)	9.0	3.6	11.8	4.3	79.4	6.0																		
[27]	(peripheral blood) Macrophages (high fat diet)	10.3	3.5	13.5	4.2	76.6	1.7																		
[28]	(ovarian) PEO1 ovarian cancer cell line (CD90-)	54.0	3.0	19.3	0.5																				
[28]	(ovarian) PEO1 ovarian cancer cell line (CD90+)	39.1	3.0	25.7	1.1																				
[29]	(peripheral blood) PBMCs (control)	0.0	0.0	33.5	7.4	5.5	10.5	58.7	7.4	90.0	8.3	48.9	6.6	58.8	7.3	57.7	10.5	44.9	12.4						
[29]	(peripheral blood) PBMCs (diabetes mellitus type 2)	0.0	0.0	33.5	6.0	0.0	0.0	65.3	7.1	87.3	8.3	59.9	6.3	65.2	7.1	55.2	9.8	59.9	6.4						
[30]	(lung) Normal primary human bronchial epithelial (NHBE) cells	73.4	6.7	20.7	5.2	31.6	4.4	60.1	5.3	8.9	3.1	12.4	5.7	-13.3		-11.8		-19.2							
[31]	(peripheral blood) Human platelets	37.4	5.3			32.0	9.7	60.8				58.8		23.5	2.4			26.8	8.5						
[32]	(fibroblast) Mouse Embryonic Fibroblasts (MEFs) MEFs	49.4	5.2			38.8	5.7	56.0				63.3		6.5	8.7			24.6	2.4						

**[32]**	**(fibroblast) Mouse Embryonic Fibroblasts (MEFs)**					44.0						99.3						55.2							
**[33]**	**(stem cells) Human MSCs (not stimulated)**	39.0	5.6	25.5	5.3	38.5	6.3	28.6	7.5	24.5	7.0	44.8	6.1	-10.4		-0.9		6.2							
**[33]**	**(stem cells) Human MSCs (stimulated)**	36.1	6.7	24.3	4.6	50.2	6.2	35.5	4.9	18.0	6.7	58.4	5.0	-0.6		-6.3		8.2							
**[34]**	**(kidney) Primary podocytes**	14.0	2.8	4.5	3.7	70.8	6.5																		
**[35]**	**(placenta) Human syncytiotrophoblast**	31.8	15.9	35.6	19.2	34.6	18.3	49.8	9.3	49.9	28.5	51.2	6.6	18.8	14.5	14.4	23.6	18.2	14.1						
**[35]**	**(placenta) Human syncytiotrophoblast (obese)**	34.4	18.4	32.2	26.0	32.0	15.1	49.6	10.7	40.0	27.0	49.8	10.9	15.4	14.6	11.9	11.5	18.5	11.4						
**[35]**	**(placenta) Human syncytiotrophoblast (diabetic)**	37.0	15.7	28.9	15.0	40.1	14.5	52.9	6.9	36.3	7.4	56.6	9.5	16.9	13.4	8.1	12.2	16.3	10.7						
**[36]**	**(liver) HepG2 cells (control)**					33.9	7.7					53.1						19.3	6.4						
**[36]**	**(liver) HepG2 cells (thyroid hormone stimulated)**					80.9	8.0					83.8						3.0	3.0						
**[37]**	**(heart) Adult ventricular cardiomyocyte cells (AC16)**					23.4	2.9					25.0	20.7					1.6							
**[38]**	**(lung) A549**	62.2	6.6	4.6	3.5	49.9	6.4	83.5	4.5	-5.9	4.0	41.4	6.4	21.3		-10.5		-8.4							
**[39]**	**(liver) Huh7.5 (FBS)**	28.7		36.9		32.8																			
**[39]**	**(liver) Huh7.5 (human serum)**	20.0		31.3		55.0																			
**[40]**	**(lung) A549**	30.8		39.0		29.7																			
**[40]**	**(lung) A549 (radiotherapy resistant)**	55.9		27.7		15.5																			
**[41]**	**(breast) BT549**	17.8	2.1	5.0	2.9	13.7	2.0	17.4	4.8	18.2	2.8	28.2	2.1	-0.3		13.3		14.5							
**[41]**	**(breast) ESH-172**	10.4	4.2	9.5	4.5	10.9	4.3	19.1	3.3	16.4	5.7	34.1	6.3	8.7		6.9		23.2							
**[41]**	**(breast) MDA-MB-175-VII**	8.0	3.0	13.3	2.1	16.9	1.3	25.3	6.6	23.5	7.6	13.3	0.4	17.3		10.2		-3.6							
**[41]**	**(breast) Hs578T**	11.2	5.2	22.2	1.6	18.7	4.0	21.2	5.6	3.2	3.0	23.6	5.5	10.0		-19.1		4.9							
**[42]**	**(fibroblast) Primary human fibroblasts (not senescent)**	44.8	4.1	36.7	5.8	18.5	3.2																		
**[42]**	**(fibroblast) Primary human fibroblasts (senescent)**	44.4	4.3	26.2	5.8	29.4	9.5																		
**[43]**	**(brain) Human primary astrocytes**	29.0	3.3	25.1	2.6	15.1	2.0	72.3	7.8	53.2	3.2	58.6	3.6	46.2	6.8	42.9	4.7	46.0	3.4						
**[43]**	**(brain) Human primary astrocytes (glucose deprivation adapted)**	31.6	2.2	20.0	2.7	21.1	2.9	74.4	6.0	50.6	2.5	52.4	4.1	40.2	3.7	53.9	3.5	38.6	3.4						
**[44]**	**(skin) Patient-derived melanoma cells Mel2400 (control)**	0.0	0.0	34.4	0.4	7.7	12.4																		
**[44]**	**(skin) Patient-derived melanoma cells Mel2400 (ALDOA-overexpressing)**	2.6	7.5	28.8	5.3	14.8	7.5																		
**[45]**	**(brian) SK-N-AS neuroblastoma cells**	46.0	1.5	13.4	3.6	48.6	2.4	52.0	2.2	33.3	1.2	55.1	1.1	6.2		19.8		11.8							
**[46]**	**(breast) SUM44**	76.6		15.4		7.5		70.5		25.7		19.2		-6.1		10.2		11.6							
**[47]**	**(ovarian) Cisplatin-resistant human ovarian carcinoma A2780/CP70**	42.3	2.1	69.6	4.2	53.4	4.4	44.1	6.5	42.5	4.7	38.2	6.2	15.0	6.7	-30.4	3.7	-18.2	6.4						
**[48]**	**(peripheral blood) Bone marrow derived dendritic cells (DC)**	48.2	16.0	35.3	16.2	51.5	21.4	52.1	18.0	36.7	15.0	54.2	16.4	6.2	13.0	2.3	15.9	6.6	8.5						

**[49]**	**(cervix) HeLa**	35.9	3.3	25.8	8.5	25.6	6.3																		
**[50]**	**(fibroblast) MEFs**	15.0	2.4	16.3	5.0	23.3	2.2							0.6	0.1	0.8	4.6	0.8	3.0						
**[51]**	**(breast) MCF7**	69.2	4.1	16.7	6.2	3.3	3.3							27.2	4.1	13.2	6.3	1.4	4.7						

Table S3: Summary data from Seahorse XF mitochondrial fuel flexibility assays. We have explored the Agilent Cell Analysis Publication Database [52; 53] for publications using the Mito Fuel Flex/Substrate Oxidation Assays in the period of 2016-2021. Furthermore, Google Scholar database was manually searched with the following syntax to improve results: “Dependency” “Seahorse”; “Flexibility” “Seahorse”; “Capacity” “Seahorse” (last 5 years). Publications were screened for data in the text and, where not available, percentages were estimated using ImageJ as described in Table S1. Seahorse XF Mito Fuel Flex and Substrate Oxidation Assays involve real-time measurement of OCR with simultaneous injections of single pathway inhibitors (etomoxir, UK5099, or BPTES) to independently block glucose oxidation (entry of pyruvate into the mitochondria via MPC), glutamine oxidation (glutaminolysis via GLS) and long-chain fatty acid oxidation (palmitate processing via CPT1). This step is then followed by 3 sequential injections of oligomycin, FCCP, and rotenone/antimycin A to calculate: basal oxidation of any given substrate (dependency), maximal oxidation (flexibility) and substrate oxidation capacity (dependency + flexibility). Empty cells indicate data was not given or could not be estimated.

References:

[1] Pasquale, V., Ducci, G., Campioni, G., Ventrici, A., Assalini, C., Busti, S., et al., 2020. Profiling and Targeting of Energy and Redox Metabolism in Grade 2 Bladder Cancer Cells with Different Invasiveness Properties. 9(12):2669.

[2] Cuyàs, E., Fernández-Arroyo, S., Alarcón, T., Lupu, R., Joven, J., Menendez, J.A.J.O., 2016. Germline BRCA1 mutation reprograms breast epithelial cell metabolism towards mitochondrial-dependent biosynthesis: Evidence for metformin-based “starvation” strategies in BRCA1 carriers. 7(33):52974.

[3] Held, N.M., Kuipers, E.N., van Weeghel, M., van Klinken, J.B., Denis, S.W., Lombès, M., et al., 2018. Pyruvate dehydrogenase complex plays a central role in brown adipocyte energy expenditure and fuel utilization during short-term beta-adrenergic activation. 8(1):1-12.

[4] Enyart, D.S., Crocker, C.L., Stansell, J.R., Cutrone, M., Dintino, M.M., Kinsey, S.T., et al., 2020. Low-dose caffeine administration increases fatty acid utilization and mitochondrial turnover in C2C12 skeletal myotubes. 8(1):e14340.

[5] Kim, E.J., Lee, M., Kim, D.Y., Kim, K.I., Yi, J.Y.J.C., 2019. Mechanisms of energy metabolism in skeletal muscle mitochondria following radiation exposure. 8(9):950.

[6] Gorski, D.J., Petz, A., Reichert, C., Twarock, S., Grandoch, M., Fischer, J.W.J.S.r., 2019. Cardiac fibroblast activation and hyaluronan synthesis in response to hyperglycemia and diet-induced insulin resistance. 9(1):1-11.

[7] Schcolnik-Cabrera, A., Chavez-Blanco, A., Dominguez-Gomez, G., Juarez, M., Lai, D., Hua, S., et al., 2020. The combination of orlistat, lonidamine and 6-diazo-5-oxo-L-norleucine induces a quiescent energetic phenotype and limits substrate flexibility in colon cancer cells. 20(3):3053-3060.

[8] Nacarelli, T., Azar, A., Altinok, O., Orynbayeva, Z., Sell, C.J.G., 2018. Rapamycin increases oxidative metabolism and enhances metabolic flexibility in human cardiac fibroblasts. 40(3):243-256.

[9] Steyn, F.J., Li, R., Kirk, S.E., Tefera, T.W., Xie, T.Y., Tracey, T.J., et al., 2020. Altered skeletal muscle glucose–fatty acid flux in amyotrophic lateral sclerosis. 2(2):fcaa154.

[10] Vitiello, G.A., Medina, B.D., Zeng, S., Bowler, T.G., Zhang, J.Q., Loo, J.K., et al., 2018. Mitochondrial inhibition augments the efficacy of imatinib by resetting the metabolic phenotype of gastrointestinal stromal tumor. 24(4):972-984.

[11] Hoffman, D.L., 2019. Mitochondrial Fuel Flexibility Assessment in a Tumor-Relevant Model. Future Science.

[12] Wang, L., Yu, Z., Ren, S., Song, J., Wang, J., Du, G.J.B.e.B.A.-G.S., 2018. Metabolic reprogramming in colon cancer reversed by DHTS through regulating PTEN/AKT/HIF1α mediated signal pathway. 1862(10):2281-2292.

[13] Chatterjee, A., Seyfferth, J., Lucci, J., Gilsbach, R., Preissl, S., Böttinger, L., et al., 2016. MOF acetyl transferase regulates transcription and respiration in mitochondria. 167(3):722-738. e723.

[14] MacVicar, T., Ohba, Y., Nolte, H., Mayer, F.C., Tatsuta, T., Sprenger, H.-G., et al., 2019. Lipid signalling drives proteolytic rewiring of mitochondria by YME1L. 575(7782):361-365.

[15] Liao, M., Liao, W., Xu, N., Li, B., Liu, F., Zhang, S., et al., 2019. LncRNA EPB41L4A-AS1 regulates glycolysis and glutaminolysis by mediating nucleolar translocation of HDAC2. 41:200-213.

[16] Fang, B., Zhang, M., Ge, K., Xing, H., Ren, F.J.J.o.d.s., 2018. α-Lactalbumin-oleic acid complex kills tumor cells by inducing excess energy metabolism but inhibiting mRNA expression of the related enzymes. 101(6):4853-4863.

[17] Kors, L., Rampanelli, E., Stokman, G., Butter, L.M., Held, N.M., Claessen, N., et al., 2018. Deletion of NLRX1 increases fatty acid metabolism and prevents diet-induced hepatic steatosis and metabolic syndrome. 1864(5):1883-1895.

[18] Moriyama, T., Kiyonaga, N., Ushikai, M., Kawaguchi, H., Horiuchi, M., Kanmura, Y.J.O.J.o.A., 2018. Effects of L-carnitine on propofol-induced inhibition of free fatty acid metabolism in fasted rats and in vitro. 8(05):147.

[19] Beaudin, S., Welsh, J.J.E., 2017. 1, 25-Dihydroxyvitamin D regulation of glutamine synthetase and glutamine metabolism in human mammary epithelial cells. 158(12):4174-4188.

[20] Yetkin-Arik, B., Vogels, I., Neyazi, N., Van Duinen, V., Houtkooper, R., Van Noorden, C., et al., 2019. Endothelial tip cells in vitro are less glycolytic and have a more flexible response to metabolic stress than non-tip cells. 9(1):1-17.

[21] Duman, C., Yaqubi, K., Hoffmann, A., Acikgöz, A.A., Korshunov, A., Bendszus, M., et al., 2019. Acyl-CoA-binding protein drives glioblastoma tumorigenesis by sustaining fatty acid oxidation. 30(2):274-289. e275.

[22] Yao, H., Gong, J., Peterson, A.L., Lu, X., Zhang, P., Dennery, P.A.J.A.j.o.r.c., et al., 2019. Fatty acid oxidation protects against hyperoxia-induced endothelial cell apoptosis and lung injury in neonatal mice. 60(6):667-677.

[23] Podrini, C., Rowe, I., Pagliarini, R., Costa, A.S., Chiaravalli, M., Di Meo, I., et al., 2018. Dissection of metabolic reprogramming in polycystic kidney disease reveals coordinated rewiring of bioenergetic pathways. 1(1):1-14.

[24] Vogel, F.C., Bordag, N., Zügner, E., Trajkovic-Arsic, M., Chauvistré, H., Shannan, B., et al., 2019. Targeting the H3K4 demethylase KDM5B reprograms the metabolome and phenotype of melanoma cells. 139(12):2506-2516. e2510.

[25] Gao, Y., Vidal-Itriago, A., Kalsbeek, M.J., Layritz, C., García-Cáceres, C., Tom, R.Z., et al., 2017. Lipoprotein lipase maintains microglial innate immunity in obesity. 20(13):3034-3042.

[26] Anderson, C.C., Aivazidis, S., Kuzyk, C.L., Jain, A., Roede, J.R.J.T.S., 2018. Acute maneb exposure significantly alters both glycolysis and mitochondrial function in neuroblastoma cells. 165(1):61-73.
[27] Baardman, J., Verberk, S.G., Prange, K.H., van Weeghel, M., van der Velden, S., Ryan, D.G., et al., 2018. A defective pentose phosphate pathway reduces inflammatory macrophage responses during hypercholesterolemia. 25(8):2044-2052. e2045.
[28] Kitajima, S., Lee, K.L., Hikasa, H., Sun, W., Huang, R.Y.-J., Yang, H., et al., 2017. Hypoxia-inducible factor-1α promotes cell survival during ammonia stress response in ovarian cancer stem-like cells. 8(70):114481.
[29] Nicholas, D.A., Proctor, E.A., Agrawal, M., Belkina, A.C., Van Nostrand, S.C., Panneerseelan-Bharath, L., et al., 2019. Fatty acid metabolites combine with reduced β oxidation to activate Th17 inflammation in human type 2 diabetes. 30(3):447-461. e445.
[30] Smallwood, H.S., Duan, S., Morfouace, M., Rezinciuc, S., Shulkin, B.L., Shelat, A., et al., 2017. Targeting metabolic reprogramming by influenza infection for therapeutic intervention. 19(8):1640-1653.
[31] Aibibula, M., Naseem, K., Sturmey, R.J.J.o.T., Haemostasis, 2018. Glucose metabolism and metabolic flexibility in blood platelets. 16(11):2300-2314.
[32] Lue, H.-w., Podolak, J., Kolahi, K., Cheng, L., Rao, S., Garg, D., et al., 2017. Metabolic reprogramming ensures cancer cell survival despite oncogenic signaling blockade. 31(20):2067-2084.
[33] Jitschin, R., Böttcher, M., Saul, D., Lukassen, S., Bruns, H., Loschinski, R., et al., 2019. Inflammation-induced glycolytic switch controls suppressivity of mesenchymal stem cells via STAT1 glycosylation. 33(7):1783-1796.
[34] Brinkkoetter, P.T., Bork, T., Salou, S., Liang, W., Mizi, A., Özel, C., et al., 2019. Anaerobic glycolysis maintains the glomerular filtration barrier independent of mitochondrial metabolism and dynamics. 27(5):1551-1566. e1555.
[35] Wang, Y., Bucher, M., Myatt, L.J.T.J.o.C.E., Metabolism, 2019. Use of Glucose, Glutamine, and Fatty Acids for Trophoblast Respiration in Lean Women, Women With Obesity, and Women With Gestational Diabetes. 104(9):4178-4187.
[36] Singh, B.K., Sinha, R.A., Tripathi, M., Mendoza, A., Ohba, K., Sy, J.A., et al., 2018. Thyroid hormone receptor and ERRα coordinately regulate mitochondrial fission, mitophagy, biogenesis, and function. 11(536).
[37] Zhang, J., Yu, L., Xu, Y., Liu, Y., Li, Z., Xue, X., et al., 2018. Data on long noncoding RNA upregulated in hypothermia treated cardiomyocytes protects against myocardial infarction through improving mitochondrial function. 17:610-625.
[38] Conroy, L.R., Lorkiewicz, P., He, L., Yin, X., Zhang, X., Rai, S.N., et al., 2020. Palbociclib treatment alters nucleotide biosynthesis and glutamine dependency in A549 cells. 20(1):1-12.
[39] Steenbergen, R., Oti, M., Ter Horst, R., Tat, W., Neufeldt, C., Belovodskiy, A., et al., 2018. Establishing normal metabolism and differentiation in hepatocellular carcinoma cells by culturing in adult human serum. 8(1):1-14.
[40] Lee, D.E., Alhallak, K., Jenkins, S.V., Vargas, I., Greene, N.P., Quinn, K.P., et al., 2018. A radiosensitizing inhibitor of HIF-1 alters the optical redox state of human lung cancer cells in vitro. 8(1):1-10.
[41] Martin, S.D., McGee, S.L.J.C., metabolism, 2019. A systematic flux analysis approach to identify metabolic vulnerabilities in human breast cancer cell lines. 7(1):1-14.
[42] Kim, S.-J., Mehta, H.H., Wan, J., Kuehnemann, C., Chen, J., Hu, J.-F., et al., 2018. Mitochondrial peptides modulate mitochondrial function during cellular senescence. 10(6):1239.
[43] Potter, P.G.W., Walker, J.M.V., Robb, J.L., Chilton, J.K., Williamson, R., Randall, A.D., et al., 2019. Basal fatty acid oxidation increases after recurrent low glucose in human primary astrocytes. 62(1):187-198.
[44] Cascone, T., McKenzie, J.A., Mbofung, R.M., Punt, S., Wang, Z., Xu, C., et al., 2018. Increased tumor glycolysis characterizes immune resistance to adoptive T cell therapy. 27(5):977-987. e974.
[45] Kuzyk, C.L., Anderson, C.C., Roede, J.R.J.C., science, t., 2020. Simvastatin induces delayed apoptosis through disruption of glycolysis and mitochondrial impairment in neuroblastoma cells. 13(3):563-572.
[46] Martin, L.-A., Ribas, R., Simigdala, N., Schuster, E., Pancholi, S., Tenev, T., et al., 2017. Discovery of naturally occurring ESR1 mutations in breast cancer cell lines modelling endocrine resistance. 8(1):1-15.
[47] Pathak, R.K., Wen, R., Kolishetti, N., Dhar, S.J.M.c.t., 2017. A prodrug of two approved drugs, cisplatin and chlorambucil, for chemo war against cancer. 16(4):625-636.
[48] Rezinciuc, S., Bezavada, L., Bahadoran, A., Duan, S., Wang, R., Lopez-Ferrer, D., et al., 2020. Dynamic metabolic reprogramming in dendritic cells: An early response to influenza infection that is essential for effector function. 16(10):e1008957.
[49] Salewskij, K., Rieger, B., Hager, F., Arroum, T., Duwe, P., Villalta, J., et al., 2020. The spatio-temporal organization of mitochondrial F1FO ATP synthase in cristae depends on its activity mode. 1861(1):148091.
[50] Pacelli, C., Adipietro, I., Malerba, N., Squeo, G.M., Piccoli, C., Amoresano, A., et al., 2020. Loss of Function of the Gene Encoding the Histone Methyltransferase KMT2D Leads to Deregulation of Mitochondrial Respiration. 9(7):1685.
[51] Kam, Y., Romero, N., Swain, P., Dranka, B.P., 2017. Characterization of fuel dependencies in multidrug resistant breast cancer cells, Presented at the American Association of Cancer Researchers Annual Meeting.
[52] Cell, A.S.X.H.T., Kit, A.A., 2020. Agilent Seahorse XF Hu T Cell Activation Assay Kit.
[53] Leung, D.T., Chu, S., 2018. Measurement of oxidative stress: mitochondrial function using the seahorse system. Preeclampsia. Springer, p. 285-293.
